# Monitoring of singlet oxygen generation of a novel Schiff-base substituted silicon phthalocyanines by sono-photochemical studies and in vitro activities on prostate cancer cell

**DOI:** 10.1007/s00775-024-02055-z

**Published:** 2024-05-10

**Authors:** Hiba Messaoudi, Göknur Yaşa Atmaca, Ayşegül Türkkol, Mehmet Dinçer Bilgin, Ali Erdoğmuş

**Affiliations:** 1 Department of Chemistry, Yildiz Technical University, 34210 Esenler, Istanbul Turkey; 2 Faculty of Medicine, Department of Biophysics, Aydın Adnan Menderes University, 09010 Aydın, Turkey; 3 Health Biotechnology Joint Research and Application Center of Excellence, 34220 Istanbul, Turkey

**Keywords:** Sono-photodynamic therapy, Singlet oxygen, Prostate cancer, PC3 cell line

## Abstract

This study demonstrates the potential of sono-photodynamic therapy as an effective approach for enhancing singlet oxygen generation using the synthesized Schiff-base diaxially substituted silicon phthalocyanines. In photochemical studies, the singlet oxygen quantum yields (**Φ**_**∆**_) were determined as 0.43 for **Si1a**, 0.94 for **Q-Si1a**, 0.58 for **S-Si1a**, and 0.49 for **B-Sia1**. In sono-photochemical studies, the Φ_∆_ values were reached to 0.67 for **Si1a**, 1.06 for **Q-Si1a**, 0.65 for **S-Si1a**, and 0.67 for **B-Sia1**. In addition, this study demonstrates the therapeutic efficacy of phthalocyanines synthesized as sensitizers on the PC3 prostate cancer cell line through in vitro experiments. The application of these treatment modalities exhibited notable outcomes, leading to a substantial decrease in cell viability within the PC3 prostate cancer cell line. These findings highlight the potential of utilizing these synthesized phthalocyanines as promising therapeutic agents for prostate cancer treatment.

**Graphical Abstract:**

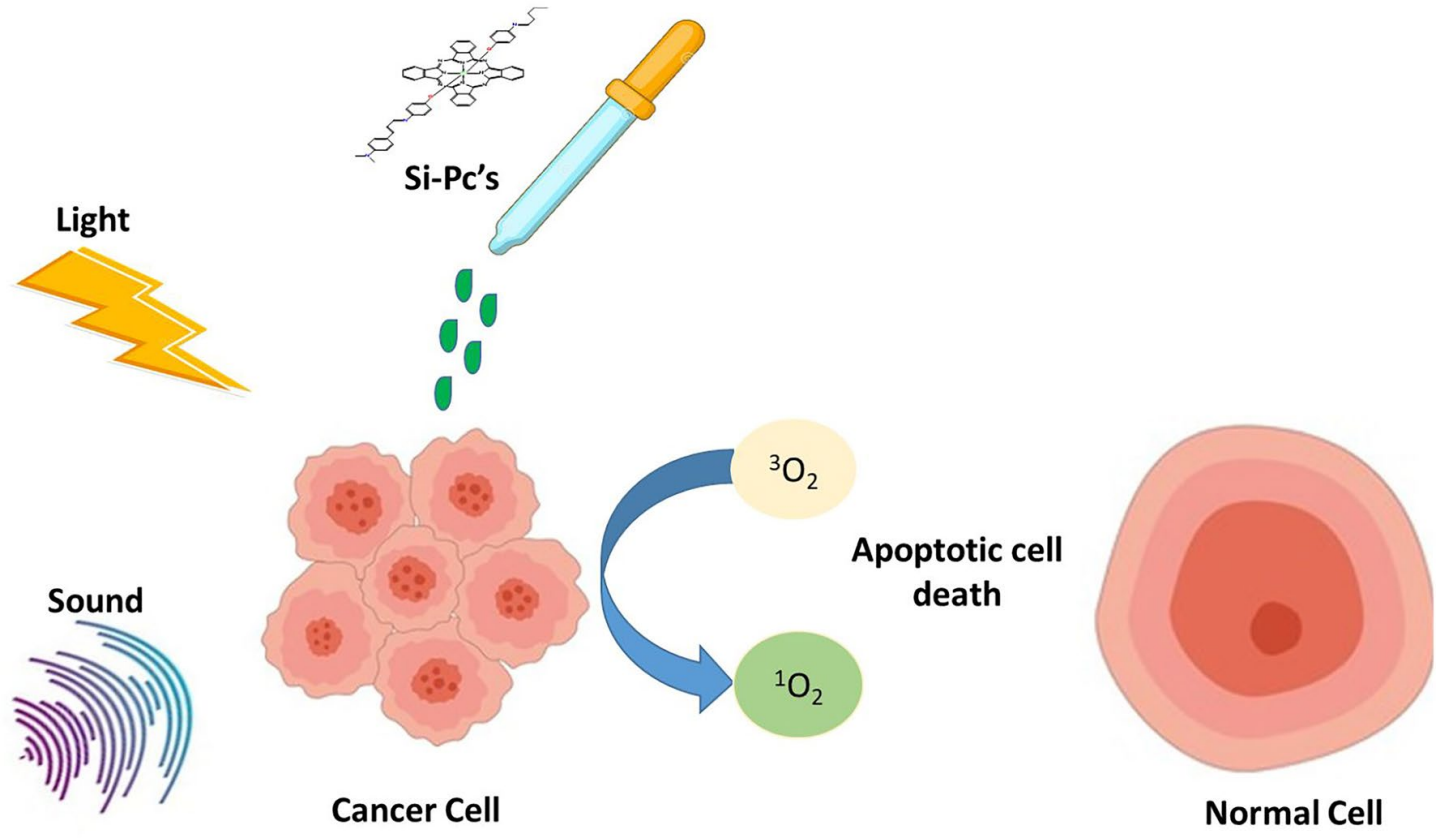

**Supplementary Information:**

The online version contains supplementary material available at 10.1007/s00775-024-02055-z.

## Introduction

Prostate cancer is a significant global health concern, with its incidence steadily increasing. It currently stands as the second most commonly diagnosed cancer among men worldwide and ranks fifth as a cause of cancer-related deaths [1]. Conventional treatment options for prostate cancer include surgery, radiation therapy, hormonal therapy, and chemotherapy. While these approaches have demonstrated efficacy, there is a pressing need to explore new therapeutic strategies that can further improve patient outcomes. Photodynamic therapy (PDT) is a treatment that appeared in the early 1980s but has recently gained popularity for various conditions such as acne, psoriasis or acute macular degeneration. First used for the treatment of skin cancers, then quickly extended to cancers of the prostate and the respiratory system, it is based on the combined action of three harmless components when taken separately: oxygen, photosensitive agents and light. Photodynamic therapy thus kills cancer cells by apoptosis, by producing reactive oxygen species (ROS) [2–4]. Photosensitive agents are introduced at the site of the tumor in the form of a cream for skin cancers or intravenously near the tumor for other cancers. These agents are absorbed within hours by cancer cells (preferably accumulate in rapidly growing cells) and then are activated by a light source of a specific wavelength, which causes them to react with the cell oxygen to form free radicals [5]. Because of the light penetrating shallowly into the tissues, some authors have used these sensitizing substances with ultrasound, which can penetrate several centimeters into the body: this is sound therapy [6].Sonodynamic therapy (SDT) involves the systemic administration of a non-toxic (sono-sensitizing) agent that accumulates in tumor cells. Then, exposure to low-intensity ultrasound activates the drug, which induces targeted apoptosis. Preclinical studies indicate that SDT is a promising approach for the non-invasive treatment of lesions that are difficult to access, while sparing non-target tissue [7, 8]. A combination of both PDT and SDT methods named “sono-photodynamic therapy (SPDT)” can provide a better therapeutic approach, using ultrasound of a particular frequency and light of a particular wavelength together to stimulate the sensitizer [7–11]. Therefore, the choice of an efficient sensitizer for SDT, PDT, and SPDT is of a huge challenge. A good sensitizer is qualified to have stable chemical composition, a high excision rate from normal tissues, and must be nearby zero toxicity [12]. Silicon-phthalocyanine being known as the second generation of sensitizers, is distinguished by the fact that it has two extra axial bonds which proffer it reduced aggregation in most solutions compared to its phthalocyanine counterparts [13]. SiPc's are widely used for photochemical and sono-photochemical applications due to their exceptional near-infrared region absorption properties. SiPc's are effective sono/photosensitizers that can cause cellular toxicity with light and ultrasound, making them a preferred anti-cancer agent. Therefore, SiPc's have a high singlet oxygen generation ability, making them a popular choice as sensitizers in PDT and SPDT [12, 14, 15]. The purpose of this work is the evaluation of the cytotoxic effect of light (PDT) and ultrasound combined (SPDT) on cancer cells, after the sensitization of four novel synthesized axially Schiff base di-substituted silicon (IV) phthalocyanine compounds, by the generation of singlet oxygen. The findings of this research may contribute to the development of more effective therapeutic approaches for prostate cancer.

## Experimental

Supplementary information assembles the equipment, materials, and all theoretical parameters utilized in the study.

### Synthesis

#### Synthesis of 4-[(E)-{[4-(dimethylamino) phenyl] methyldien} amino] phenol (1a)

4(dimethylamino)benzaldehyde (149 mg, 1 mmol) and 5 mL of dry ethanol were introduced to a two-necked flask, after that a solution of 4-aminophenol (112 mg, 1 mmol) in 5 mL of dry ethanol was added in portions. Followed by that, 5 drops of glacial acetic acid were added and stirred continuously at 78 ºC under reflux. three and a half hours later, the yellow-colored substance formed was filtered off. It was washed with cold methanol and dried. Finally, the pure compound was dried in vacuum. Yield: %95, **FT-IR** νmax/cm^−1^: 3061 (Ar–CH), 2988–2819 (Aliph. -CH), 1606 (C = N), 1586, 1534, 1504 (C=C). ^**1**^**H-NMR** (d-DMSO), (δ:ppm): 9.32 (s, 1 Ar-OH), 8.39 (s, 1 HC = N), 7.70 (d, J ≈ 8.8 Hz; 2 Ar–H), 7.09 (d, J ≈ 8.8 Hz; 2 Ar–H), 6.78–6.75 (m, 4 Ar–H), 3.33 (s, 6H, 2CH_3_). **MALDI-TOF–MS**, (m/z): Calculated. 240.30; Found. 240.937 [M]^+^. **Elemental analysis**: Calculated. C, 74.97; H, 6.71; N, 11.66; Found: C, 74,65; H, 6.69; N, 11.62.

#### Synthesis of 4-[(E)-{[4-(dimethylamino) phenyl] methylidene} amino] phenol di-substituted Silicon Phthalocyanine (Si1a)

Compound **1a** (160 mg, 0.65 mmol) was dissolved in 10 mL of toluene and added to SiPcCl_2_ (200 mg, 0.33 mmol). Anhydrous NaH (16 mg, 0.65 mmol) was added to this mixture after 10 min. The prepared mixture was left to stirring under reflux at 110 °C in an argon gas atmosphere for 24 h. At the end of the reaction, the product was cooled to room temperature and the solvent was evaporated. The resulting complex was checked by thin layer chromatography (TLC) and purified by column chromatography. CHCl_3_:CH_3_OH (100:5) was used as the solvent system. Yield: 20%, **UV–Vis** (DMSO): λ_max_/ nm (log ε): 680 nm (Q band), 614 nm (Q' band), 357 nm (B-band). **FT-IR** ν_max_/cm^−1^: 2885 (Aliphatic-CH), 1602(C=N), 1522 (C=C), 1334 (N–H), 1078 (Si–O–C), and 729 (Ar–CH). ^**1**^**H-NMR** (DMSO-d6), (δ:ppm):8.37 (s, 2 H–C=N), 7.78–7.75 (m, Ar–H), 7.44–7.36 (m, Ar–H), 6.74–6.72 (m, Ar–H), 3.11 (s, 12H, 4CH_3_). **MALDI-TOF–MS**, (m/z): Calculated: 1019.19 [M + H]^+^, Found: 1020.36 [M + H]^+^. **Elemental analysis**: Calculated C, 73.06; H, 4.55; N, 16.49; Found: C, 72.54; H, 4.44; N, 16.55.

#### Synthesis of [(E)-{[4-(trimethylamino) phenyl] methylidene} amino] phenol disubstituted Phthalocyaninato Silicon Sulfate (Q-Si1a)

The compound **Si1a** (30 mg, 0.03 mmol) was placed in the Schlenk tube and 2 mL of dry DMF were added. 0.5 mL of dimethyl sulfate was added to it, the mixture was stirred at 120 °C in argon inert atmosphere for 24 h. The solution brought to room temperature and was precipitated with 30 mL of hot acetone then centrifuged. The obtained product was purified by washing with hot acetone, ethanol, ethyl acetate, dichloromethane, THF, chloroform, diethyl ether and hexane respectively. Yield: 85%, **UV–Vis** λ_max_/nm: 673 nm (Q band), 606 nm (Q' band), and 353 nm (B-band). **FT-IR** ν_max_/cm^−1^: 3065 (Ar–H), 2958–2686 (Aliph. C–H), 1608 (C=N), 1535 (C=C), 1335 (C-N), 1082 (Si–O–C), and 732 (Ar–CH). ^**1**^**H-NMR** (DMSO-d6), (δ:ppm): 9.72 (4H, d, Ar–H), 9.63 (8H, m, Ar–H), 8.46 (8H, m, Ar–H), 8.14 (2H, s, HC=N), 8.05 (8H, m, Ar–H), 7.83–7.80 (4H, m, Ar–H), 3.37 (18H, s, N-CH3). **MALDI-TOF–MS**, (m/z): Calculated: 1049.261, Found: 1129.052 [M + 2 K + 2H]^+^. **Elemental analysis**: Calculated C, 67.11; H, 4.58; N, 14.68. Found: C, 66.81; H, 4.48; N, 15.02.

#### Synthesis of 4-[(E)-{[4-(dimethyl-(3-sulfopropyl)ammonium) phenyl] methylidene} amino] phenol di-substituted Phthalocyaninato Silicon (SSi1a)

The product **Si1a** (30 mg, 0.03 mmol) was placed in the Schlenk tube and 2 mL of dry DMF were added. 0.3 mL of 1,3-propanesultone was added the mixture and stirred at an inert argon atmosphere at 70 °C for 24 h. The obtained solution brought to room temperature then was precipitated in 60 mL of dichloromethane and centrifuged. The obtained product was purified with hot acetone, ethanol, dichloromethane, diethyl ether and hexane respectively. Yield: 84%,**UV–Vis** λ_max_/nm:678 nm (Q band), 614 nm (Q’ band), 361 nm (B band). **FT-IR** ν_max_/cm^−1^: 2930 (Ar–H), 1608 (C = N), 1580, 1542 and 1504 (C=C), 1335 (C-N), 1169 (S=O), 1080 (Si–O-C), 734 (Ar–CH)**. MALDI-TOF–MS:**Calculated: 1263.48; Found: 1193.42 [M-2SO_3_ + 5H_2_O]^+^, 621.85 [C_32_H_18_N_8_O_2_Si + 2Na]^+^ .^**1**^**H-NMR**(DMSO-d6), (δ:ppm): 9.74 (8H, m, Ar–H), 9.63 (2H, m, Ar–H), 8.58 (8H, m, Ar–H), 8.46 (2H, m, Ar–H), 8.14 (2H, s, HC = N), 7.64–5.97 (12H, m, Ar–H), 3.08 (12H, s, N–CH_3_), 2.55 (4H, m, –CH_2_), 2.42 (4H, m, –CH_2_), 1.70 (4H, m, –CH_2_). **Elemental analysis**: Calculated. C, 64.64; H, 4.63; N, 13.30; Found: C, 63.75; H, 4.76; N, 13.25.

#### Synthesis of 4-[(E)-{[4-(dimethyl-(3 acetate) ammonium)phenyl]methylidene} amino]phenol di-substituted Phthalocyaninato Silicon (B-Si1a)

The product **Si1a** (40 mg, 0.039 mmol) was placed in the Schlenk tube and 3 mL of dry DMF were added. Sodium chloroacetate (24 mg, 0.2 mmol) was added to it. Argon gas was passed and stirred at 75 °C for 24 h. The solution brought to room temperature was precipitated in 50 mL of dichloromethane and centrifuged. The obtained product was purified by washing with acetone, diethylether and hexane. Yield: 81%, **UV–Vis** λmax/ nm: 679 (Q band) and 614 (Q' band) and 361 (B- band). **FT-IR** νmax/cm^−1^: 2886 (Aliph. C-H), 1604 (C=N), 1554, 1523 and 1493 (C=C), 1336 (C-N), 1165 (S=O), 1080 (Si–O–C), 731 (Ar–CH). ^**1**^**H-NMR** (DMSO-d6) (δ:ppm): 8.20 (s, 2H, 2 HC=N), 7.96–6.79 (m, 32 Ar–H), 2.89 (s, 12H, 4CH_3_),2.73 (s, 4H, 2CH_2_). **MALDI-TOF–MS,** (m/z): Calculated: 1135.28, Found: 1094.26 [M-C_2_H_2_O_2_]^+^. **Elemental analysis**: Calculated: C, 69.83; H, 4.44; N, 14.81; Found: C, 68.92; H, 4.06; N, 13.94.

### 2.2. Photophysical and sono-photochemical studies.

#### Fluorescence quantum yields

A comparative method is used to determine the fluorescence quantum yields (Φ_F_)[16] Eq. 1:

1 $${\Phi }_{F}={\Phi }_{{F}_{\left(std\right)}}\frac{F.{A}_{std}.{\eta }^{2}}{{F}_{std}.A.{\eta }_{std}^{2}}$$

where F_std_ and F are the areas under the fluorescence curves of the reference, and SiPc derivatives respectively. Astd and Aare the absorbances of the reference and sample at the excitation wavelength,${n}_{std}^{2}$ and n^2^ are the refractive indices of solvents used for the standard and the sample, respectively [17]. The standard used in the calculations was the unsubstituted ZnPc; Φ_F_ = 0.20 in DMSO[18]. The determination of the fluorescence quantum yield has been made in DMSO. All of the studied compounds beside to the samples were excited at the same relevant wavelength.

#### Singlet oxygen quantum yields (Φ_Δ_)

The photo and/or sono-generation of the singlet oxygen quantum yields were measured at open air using the comparative method with unsubstituted ZnPc as a reference. DPBF was used as a chemical quencher for the singlet oxygen formation in DMSO [19, 20] Eq. (2):

2 $${\Phi }_{\Delta }={\Phi }_{\Delta }^{std}\frac{R.{I}_{abs}^{std}}{{R}^{std}.{I}_{abs}}$$

where ${\Phi }_{\Delta }^{{\text{std}}}$ is the singlet oxygen quantum yield for the standard unsubstituted ZnPc (${\Phi }_{\Delta }^{{\text{std}}}$= 0.67) in DMSO[21]. R^std^ and R, are the DPBF photobleaching rates in the presence of the standard and samples, respectively. ${{\text{I}}}_{{\text{abs}}}^{{\text{std}}}$ and I_abs_ are the rates of light absorption by the standard and the sample respectively. Chain reactions resulting by the interaction between DPBF and the generated singlet oxygen, were reduced by lowering the quencher’s concentration to ~ 3 × 10^−5^ mol.dm^−3^ [18]. The mixtures composed of the synthesized compounds with DPBF in DMSO were irradiated with light (intensity: 7.05 × 10^15^ photons s^−1^ cm^−2^) and/or ultrasound (Frequency: 35 kHz) for PDT, and SPDT measurements. The DPBF concentration decreasing at 417 nm were monitored each 5 s using UV spectroscopy. For SPDT studies, the samples (complex + DPBF) were monitored after each 10 s irradiation (firstly 5 s by ultrasound at a frequency of 35 kHz and then 5 s by light intensity of 7.05 × 10^15^ photons s^−1^ cm^−2^).

#### Photodegradation quantum yields(Φ_d_)

The assessment of photodegradation quantum yield (Φ_d_) was conducted by employing the experimental arrangement detailed in existing literature [22]; Eq. (3):

3 $${\Phi }_{d}=\frac{\left({C}_{0}-{C}_{t}\right).V.{N}_{A}}{{I}_{abs}.S.t}$$

where “C_o_” and “C_t_” are the sample concentrations before and after irradiation respectively, “V” is the complex solution volume, “N_A_” is Avogadro’s constant “S” is the irradiated cell area, “t” is the irradiation time, "I_abs_" represents the integration of the intensity of the radiation source with the absorption of the samples. To determine Φ_d_, a light intensity of 2.38 × 10^16^ photons s^−1^ cm^−2^ was used. The photodegradation quantum yields of the investigated complexes were measured in DMSO by observing the reduction of Q-band intensity at 10-min intervals during light exposure.

### Cell culture

#### Cytotoxicity analysis

PC3 cells were cultured in RPMI 1640 (Cat No: RPMI-A, Capricorn Scientific) medium supplemented with 1% Penicillin/Streptomycin, 1% L-glutamine, and 10% fetal bovine serum (FBS)(Cat No: 16000044, Gibco). The cells were incubated at 37 °C with 5% CO_2_ and seeded into 24-well cell culture plates at a density of 10^5^ cells per well. Synthesized phthalocyanines were introduced to the cells at various concentrations (0,5-10 µM) and then incubated at 37 °C for 24 h. After a 24-h incubation period, the cell viability was assessed using the MTT (3-(4,5-dimethylthiazol-2-yl)-2,5-diphenyltetrazolium bromide) (Cat No: M6494, Invitrogen™)assay, according to the protocol described in the source [23].

#### Sono-photodynamic therapy application

PC3 cells were incubated with a concentration of 5 μM of phthalocyanines in the culture medium for 4 h in a dark environment. After this period, the cells were washed with phosphate-buffered saline (PBS)(Cat No:10010023, Gibco) and fresh medium was added. Subsequently, they were treated with ultrasound (1 MHz, 0.5 mW/cm^2^, 60 s) and/or light (0.5 mW/cm^2^, 10 min) using the BTL-5710 Sono device (Model: 5710 SONO, BTL) and/or Abet solar simulator (Model:10,500, Abet Technologies) (with long pass > 600 nm and short pass < 800 nm) respectively. After 24 h of treatment, cell viability was assessed using the MTT assay, and Hoechst 33,342(Cat No: H3570, Invitrogen) staining according to the protocol described in the source [23].

### Statistical analysis

The statistical analysis was performed using GraphPad Prism 9 software (GraphPad Prism 9 Software, San Diego, CA, USA). To determine the normal distribution of the data, the Kolmogorov–Smirnov test was conducted. For groups with a normal distribution, the One-Way ANOVA Tukey test was employed for group comparisons. In cases where the data did not exhibit a normal distribution, the Kruskal-Walli’s test was used. The significance level was set at *p* < 0.05, and significance was indicated in the graph as *p < 0.05, **p < 0.01, ***p < 0.001, and ****p < 0.0001.

## Results and discussion

### Synthesis and characterization

**Scheme 1:**
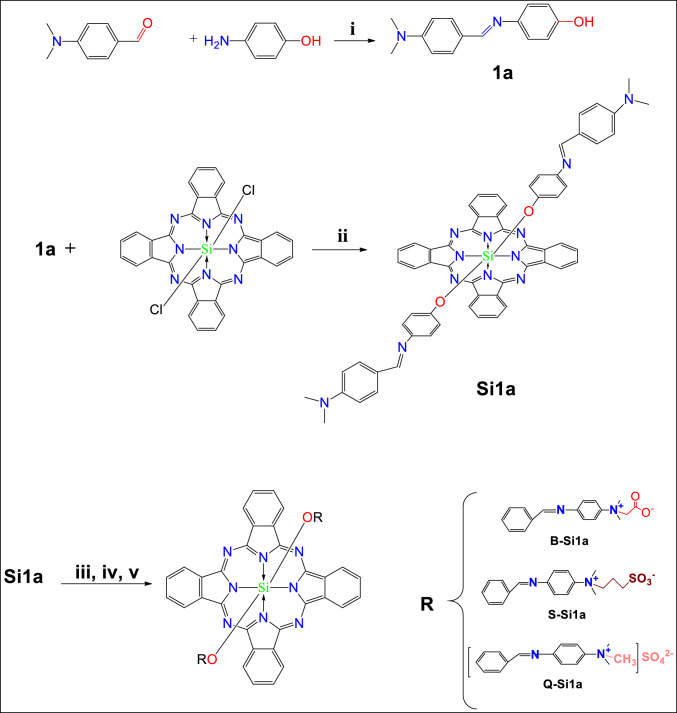
Synthesis method for axially di-substituted silicon (IV) phthalocyanines (**Si1a, Q-Si1a, S-Si1a, B-Si1a**). **i:** Glacial acetic acid, dry ethanol, 78 °C, 3.5h, Ar atm.**ii:** Toluen, NaH, 110 °C, 24h, Ar atm. **iii:** Dimethylsulfate, dry DMF, 120 °C, 24h, Ar atm. **iv:** 1,3-propansultan, dry DMF, 70 °C, 24h, Ar atm. **v:** Sodiumchloroacetate, dry DMF, 75 °C, 24h, Ar atm

Scheme1, summarizes the synthetic pathway of the axially Schiff base di-substituted silicon phthalocyanine derivatives. Ligands and complexes were characterized by UV–Vis, FT-IR, ^1^H-NMR and masse spectroscopic methods. Furthermore, their photophysical and photo/sono-chemical, properties were investigated. The spectroscopic data confirm the proposed structure of the synthesized compounds. The obtained spectral data of the synthesized complexes are given in the’supporting information’.

The **UV–Vis** spectra of the studied compounds show the presence of a sharp Q band at 680 nm for **Si1a**, 673 nm for **Q-Si1a**, 678 nm for **S-Si1a** and 679 nm for **B-Si1a**,due to the π-π* transitions[24] and 614 nm for all of**Si1a**, **S-Si1a, B-Si1a**, and 606 nm for **Q-Si1a**, (Q' band), also the presence of the B-band, which is another characteristic band originating from n- π* transitions, is observed at 357 nm for **Si1a**, 353 nm for **Q-Si1a**, and 361 nm for both **S-Si1a** and **B-Si1a**.In the **FT-IR** spectra: for the compound **1a;** the disappearance of the -NH_2_ and C = O stretching peaks, and the appearance of the C=N stretching bond at 1606 cm^−1^, beside the existence of the Ar–CH at 3061 cm^−1^, Aliphatic in the range of 2988 to 2819 cm^−1^, C = C at 1586, 1534, 1504 cm^−1^ are consistent with the expected structure. The examination of the FT-IR spectra of the SiPc compounds leads to the observation of the disappearance of the-Si-Cl [25], and –OH stretching bonds and the appearance of the characteristic Si–O–C bond for **Si1a** at 1078 cm^−1^, **Q-Si1a** at 1082 cm^−1^, **S-Si1a** at 1080 cm^−1^, and **B-Si1a** at 1080 cm^−1^, besides the S=O bond for **S-Si1a** and **B-Si1a** at 1169 cm^−1^, and 1165 cm^−1^, respectively. ^**1**^**HNMR** spectra of the synthesized compounds, **1a:** The presence of aromatic –OH at 9.32 ppm, imine proton at 8.39 ppm, aromatic protons between 7.70 and 6.75 ppm and aliphatic protons at 3.33 ppm supports the structure. The proton signal of O–H bond existing in the ligand **1a** structure disappeared in the SiPc compounds due to its replacement by the Si–O bond between the ligand and SiPc. In the SiPc compounds spectra, the H protons signals of the aromatic structure were observed as multiplet, between 7.70 and 6.75 ppm for **Si1a**, at 9.72 ppm integrating 4H, 9.63, 8.46 and 8.05 ppm integrating 24 H, also between 7.83–7.80 integrating 4H for **Q-Si1a**, 9.74 ppm for 8H, 9.63 ppm for 2H, 8.58 ppm for 8H, 8.46 for 2H, between 7.64–5.97 ppm integrating 12H for the **S-Si1a** compound. Between 7.96 and 6.79 ppm integrating 32 H for the **B-Si1a** compound. The imine proton signals were observed as singlet integrating 2H at 8.39 ppm for **Si1a**, 8.14 ppm integrating 2H **Q-Si1a**, 8.14 ppm for **S-Si1a**, and 8.20 ppm for **B-Si1a.** aliphatic protons exhibited signals as singlet at3.33 ppm for **Si1a,** at 3.37 ppm for the 18H of the N-CH_3_protons of the **Q-Si1a** compound 3.08 ppm for the 12H of N-CH_3_), 2.55 ppm for 4H, of –CH_2_), 2.42 ppm for 4H, of -CH_2_), and 1.70 ppm for the protons 4H, m, -CH_2_ for **S-Si1a.** 2.89 ppm for 2H of 4CH_**3**_ and 2.73 ppm for 4H, 2CH_2_) for **B-Si1a.** The ^**1**^**H-NMR** spectrum of the synthesized compounds supports their structures. The mass spectra of the synthesized complexes were obtained using **MALDI-TOF** spectrometry. The expected molecular ion peaks were obtained at m/z: [M]^+^: 240,937 for compound **1a**, 1020.36 [M + H]^+^ for **Si1a**, 1129.052 [M + 2 K + 2H]^+^ for **Q-Si1a.** 1193.42 [M-2SO_3_ + 5H_2_O]^+^, 621.85 [C_32_H_18_N_8_O_2_Si + 2Na]^+^ for **S-Si1a.** 1094.26 [M-C_2_H_2_O_2_]^+^for **B-Si1a**. The characterization spectra of all the synthesized compounds are illustrated in the supplementary information (S1-S19).

### Fluorescence spectra and quantum yields (Φ_F_)

**Fig. 1:**
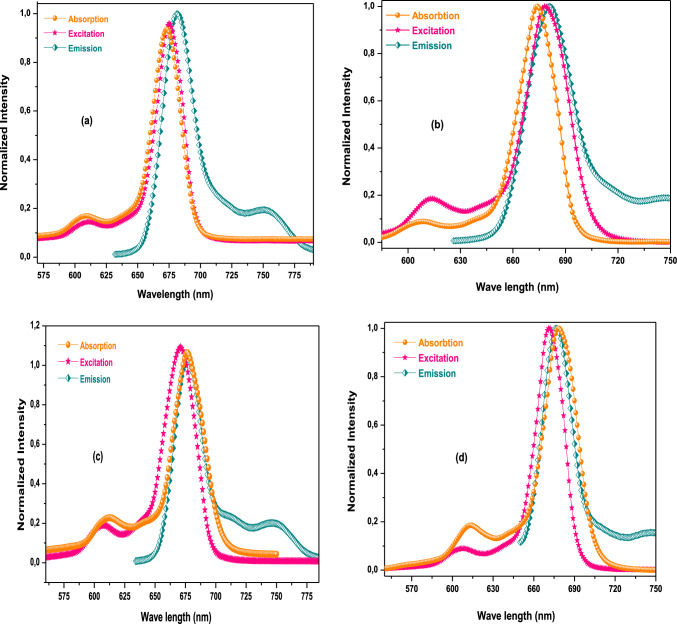
Absorption, excitation, and emission spectra of the synthesized SiPc compounds **a** **Si1a**, **b** **Q-Si1a**, **c** **S-Si1a**, **d** **B-Si1a**, in DMSO

Fluorescence quantum yield (Φ_F_) is defined as the ratio of the number of emitted photons to the number of absorbed ones. It explicates the therapeutic effect of the sensitizer for anticancer therapies. For the photochemical measurement to be held, a sensitizer should have a fluorescence behavior. Hence biocompatible DMSO was used to determine the Φ_F_ of the synthesized SiPc complexes in this study. The excitation, emission and absorption curves of the studied SiPc complexes are represented in Fig. 1. The emission and excitation spectra of the studied compounds were mirror image of each other, additionally, the adjacency of the absorption and excitation Q-Bands wavelength of each compound, can be interpreted as a result of the stability of the nuclear configuration of the base and excited states of the compounds in the studied medium (DMSO)[26–28]. The calculated values of Φ_F_ of the compounds are gathered in Table 1, 0.038 for **Si1a**, 0.16 for **Q-Si1a**, 0.27 for **S-Si1a**, and 0.088 for **B-Si1a,** which are lower than the unsubstituted silicon-phthalocyanine (SiPcCl_2_) **(**Φ_F_ = 0.44 in DMSO)[26]. These results confirm that the new axial substitutions increase notably the intersystem crossing (ISC) compared to SiPcCl_2_, thereby the singlet oxygen generation is increased and so the singlet oxygen quantum yields[12, 29].

**Table 1:** Photo-sono-physicochemical properties of axially ligated SiPc complexes in DMSO

	(Φ_F_) (10^–2^)	Φ_d_ (10^–4^)	(Φ_Δ_) PDT	(Φ_Δ_) SPDT	Reference
Si1a	0.038	2.1	0.43	0.67	This Work
Q-Si1a	0.16	2.0	0.94	1.06	
S-Si1a	0.27	1.9	0.58	0.65	
B-Si1a	0.088	5.2	0.49	0.67	
SiPcCl_2_	0.44	-	0.15	-	[19]
Unsubstituted ZnPc	0.20	-	0.67	-	
SiPc-DC	0.51	8.2	0.41		
SiPc (5)	0.015	-	0.47	-	[30]
SiPc-Ru	0.26	4.2	0.34	0.66	[26]
5	0.17	1.06	0.22	-	[31]
SiPc(3)	0.11	-	0.42	-	[27]
SiPc(4)	0.12	-	0.69	-	
SiPc-Br	0.10	2.0	0.53	0.71	[32]
1	0.10	1.2	0.50	0.81	[18]
GaPc	0.07	4.12	0.32	0.54	[10]
InPc	0.03	2.04	0.51	0.75	

### Singlet oxygen quantum yields (Φ_Δ_) for PDT and SPDT

An effective sensitizer is measured on its high ability to generate singlet oxygen. Therefore, the calculated Φ_Δ_ value that determine the singlet oxygen production capacity of a sensitizer is a major parameter[23]. In the photochemical study, the obtained singlet oxygen quantum yields values (Table 1)were 0.43 for **Si1a**, 0.94 for **Q-Si1a**, 0.58 for **S-Si1a**, and 0.49 for **B-Si1a.** Besides, the synergic effect of light irradiation all along with ultrasounds on the singlet oxygen formation increasing ability of a sensitizer has been reported in literature [33–36]. The obtained **Φ**_**Δ**_ values in this study are 0.67 for **Si1a**, 1.06 for **Q-Si1a**, 0.65 for **S-Si1a**, and 0.67 for **B-Si1a**. Overall, the complex **Q-Si1a** had the best oxygen generation, followed by the rest of the compounds having more or less the same results. The insights gained from this analysis provide valuable knowledge for future research endeavors and potential applications. Also, it is observed that the singlet oxygen quantum yield obtained in sono-photochemical measurements increased compared to photochemical measurements. The combined effect of light and ultrasound can have an impact on the stability of the molecule. Nevertheless, as depicted in Figures S20-23, no alterations were detected in the Q-band intensities of the compounds during the photochemical and sono-photochemical investigations, indicating that the silicon phthalocyanine complexes demonstrated resilience towards light and/or ultrasound. Table 1 provides a summary of previous literature studies exploring the utilization of silicon phthalocyanine as sensitizers for photochemical studies and sono-photochemical studies applications. It is evident from the table that the synthesized compounds in this study exhibit singlet oxygen quantum yields that are generally higher or comparable to those reported in previous studies. However, there is a limited amount of research available on the sono-photochemical properties of these compounds[6, 15, 26, 29, 37]. The combination of ultrasound effects with light has been shown to enhance the generation of singlet oxygen, thereby leading to a higher quantum yield. Notably, the quaternized compound **Q-Si1a** demonstrated the highest singlet oxygen quantum yield among all the compounds studied, surpassing the values reported in the existing literature.

### Photodegradation quantum yields (Φ_d_)

The sensitizer is excited by light in both PDT and SPDT methods, thus it should be stable at the applied wavelength. This part of the study deals with the stability of the synthesized compounds under light, and their capability of producing singlet oxygen without degradation during the photochemical applications. The calculated photodegradation quantum yields are listed in Table 1 and were 0.00021 for **Si1a**, 0.000192 for **Q-Si1a**, 0.00024 for **S-Si1a**, and 0.00052 for **B-Si1a** in DMSO. Compared to literature, the obtained results of the synthesized compounds have a high stability against light [38]. Thus, they are convenient to be used at PDT and SPDT as sensitizers, Figure S24, Table 1.

### MTT cytotoxicity results

The results indicate that the synthesized SiPc derivatives did not show significant cytotoxic effects up to a concentration of 5 uM after 24 h of dark incubation. Based on these findings, a concentration of 5 uM was selected as the suitable concentration for SPDT (figure S.25).

### MTT and hoechst staining results after SPDT treatment

Statistical significance was denoted by asterisks (*p < 0.05, **p < 0.01, ***p < 0.001, ****p < 0.0001) compared to the only phthalocyanine group. The data are expressed as the mean ± standard error of three independent experiments.

Fluorescence microscope images of Hoechst staining were captured at a magnification of 10 × using DAPI filter (ZEISS Microscopy, Germany). A) displays the Hoechst images of the experimental groups B) graphical presentation of cell death of groups to control. Statistical significance was denoted by asterisks (*p < 0.05, **p < 0.01, ***p < 0.001, ****p < 0.0001) compared to the only phthalocyanine group. The data are expressed as the mean ± standard error of three independent experiments.

**Fig. 2:**
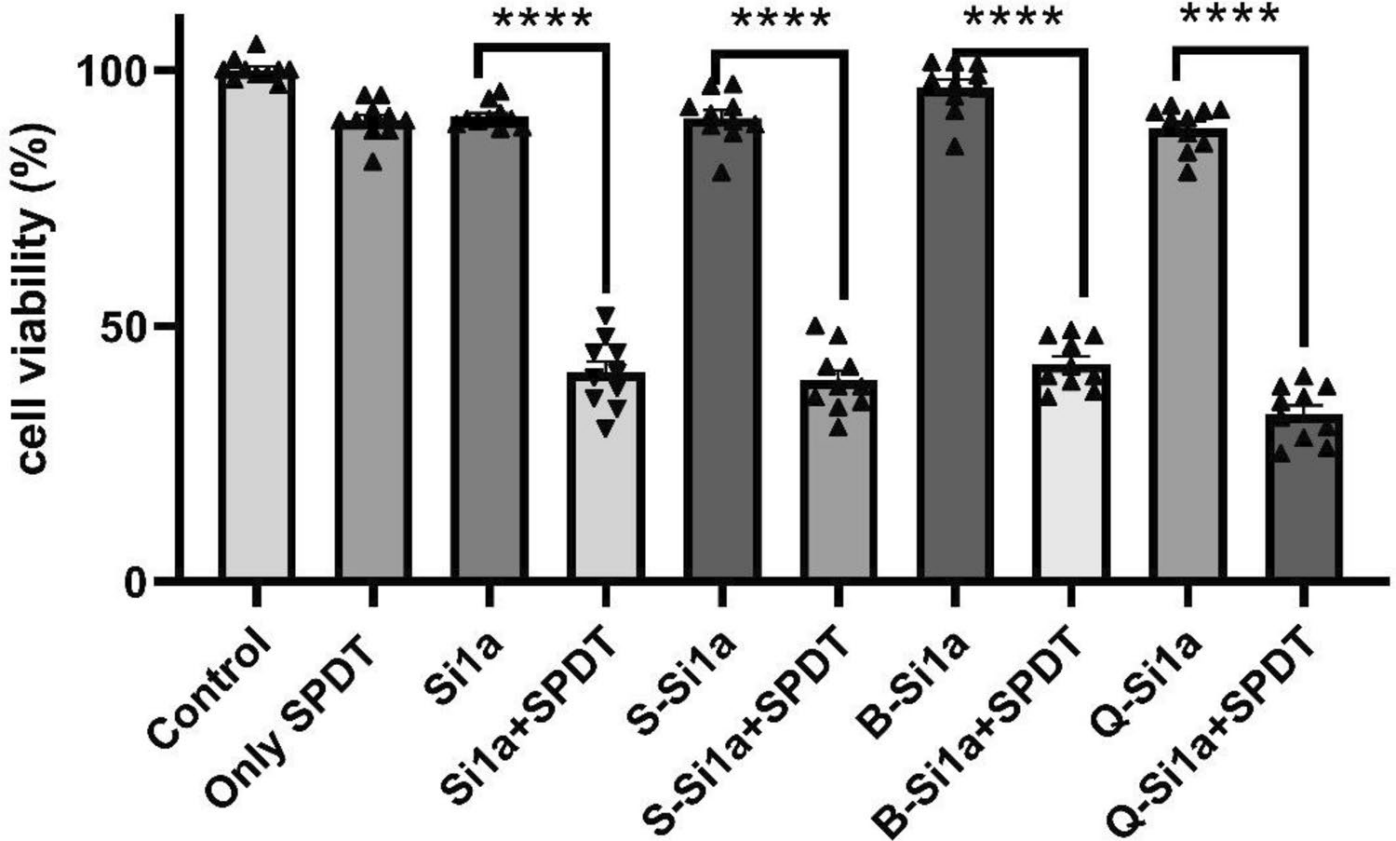
After SPDT, cell viability MTT results

**Fig. 3:**
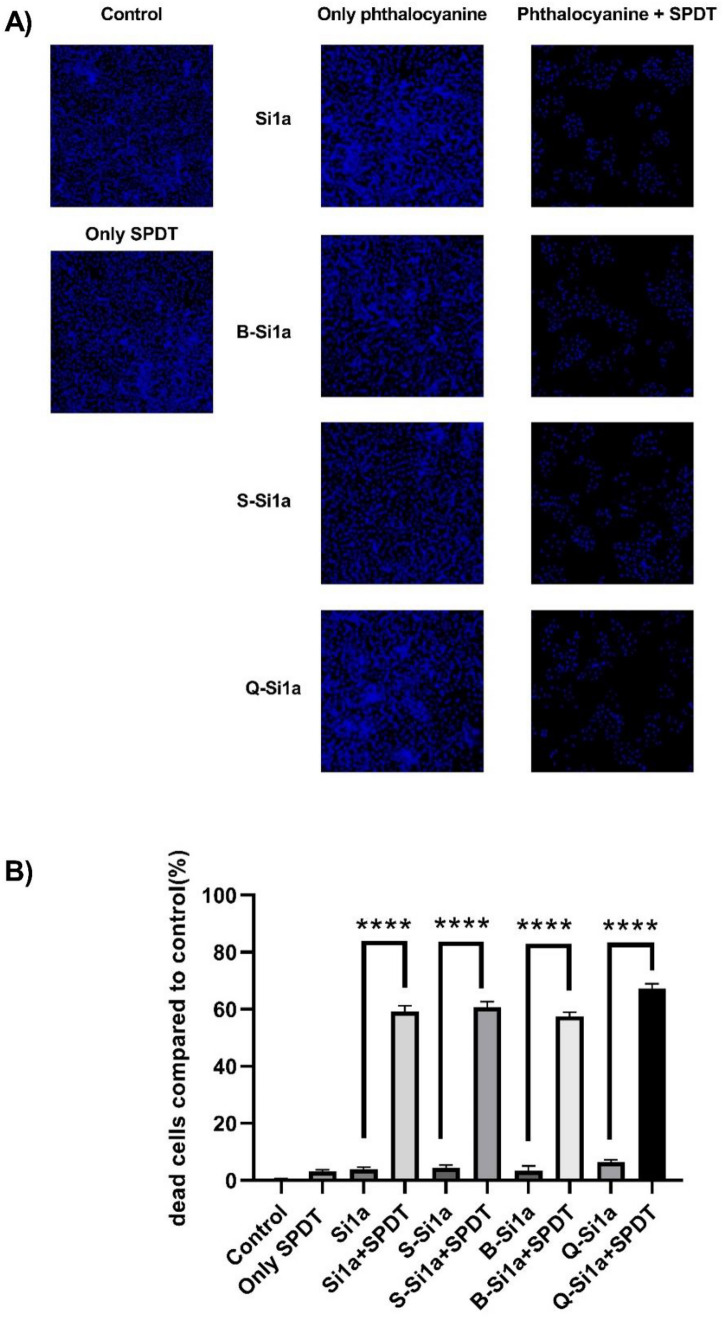
Hoechst staining cell viability results

In previous studies, it has been demonstrated that the dosage of ultrasound and light we applied does not result in significant cell death on its own[39, 40]. As observed in Fig. 2 and 3, 5 µM SiPc derivatives and the SPDT group alone did not cause any significant difference in cell viability compared to the control, however, SiPc derivatives mediated by SPDT resulted in a significant decrease in cell viability. This situation, consistent with similar studies [41, 42], demonstrates that the activation of SiPc derivatives by sound andlight leads to a decrease in cell viability and indicates their potential suitability as sensitizers for SPDT.Furthermore, the outcomes from in vitro experiments revealed that out of the synthesized **Si1a** derivatives, namely **B-Si1a,S-Si1a,** and **Q-Si1a**, it was **Q-Si1a** that displayed the most pronounced SPDT activity. This observation aligns with the recorded Φ_∆_ values.

## Conclusion

The meticulous choice of a sensitizer that generates highly efficient cytotoxic reactive oxygen, is mandatory for PDT and SPDT studies. Consequently, this study aimed to enhance the reactive oxygen generation by designing new molecules with a large conjugated system and increase their performance. Hence the SiPcCl_2_was used as a starting molecule due to its high sensitizer’s effect. The new diaxially Schiff base substituted silicon phthalocyanine compounds (**Si1a, Q**, **Si1a**, **S-Si1a**, **B-Si1a**) were synthesized and characterized. Later, their photo-physicochemical and sono-photochemical properties were investigated. When compared with SiPcCl_2_, the Φ_Δ_ values of the new molecules (0.43 for **Si1a**, 0.94 for **Q-Si1a**, 0.58 for **S-Si1a**, and 0.49 for **B-Sia1)** are higher, which leads to the conclusion that they have a more therapeutic effect due to the substituent effect. The synergic effect of ultrasounds with light was noticeable on the studied compounds compared to solely the light effect, in matter fact the Φ_Δ_ values reach (0.67 for **Si1a**, 1.06 for **Q-Si1a**, 0.65 for **S-Si1a**, and 0.67 for **B-Sia1**). Consequently, the compounds (**Si1a**, **Q-Si1a**, **S-Si1a**, **B-Si1a**) proved their photostability, and high effective sono/phototoxicity, thus their suitability to be used as sensitizers for PDT and/or SPDT applications. In vitro cell viability analyses indicated that the synthesized compounds **Si1a**, **S-Si1a**, **B-Si1a**, and **Q-Si1a** could potentially serve as sensitizer agents for SPDT. Among the four synthesized compounds, **Q-Si1a** exhibited the highest SPDT activity, which correlates with its highest **Φ**_**∆**_ value. Further investigations will be carried out to understand the anticancer mechanism of these novel Schiff-base substituted silicon phthalocyanines in prostate cancer.

### Supplementary Information

Below is the link to the electronic supplementary material.

Supplementary file 1 (PDF 1373 KB)
