# Supplementary material for: Monitoring of singlet oxygen generation of a novel Schiff-base substituted silicon phthalocyanines by sono-photochemical studies and in vitro activities on prostate cancer cell
Source: J Biol Inorg Chem. 2024 May 10;29(3):303–14. doi: 10.1007/s00775-024-02055-z (PMC11111517; doi:10.1007/s00775-024-02055-z)
Supplement: Supplementary file 1 — (PDF 1373 KB) [file 775_2024_2055_MOESM1_ESM.pdf]

Supporting Information

Monitoring of singlet oxygen generation of a novel Schiff-base substituted silicon phthalocyanines by sono-photochemical studies and in vitro activities on prostate cancer cell

Hiba MESSAOUDI[1], Göknur YAŞA ATMACA[1],Ayşegül TÜRKKOL[2], Mehmet Dinçer BİLGİN[2], Ali ERDOĞMUŞ[1*].

[1.] Department of Chemistry, Yildiz Technical University, 34210 Esenler, Istanbul, Turkey
[2.] Aydın Adnan Menderes University, Faculty of Medicine, Department of Biophysics, 09010, Aydın, Turkey
[3.] Health Biotechnology Joint Research and Application Center of Excellence, 34220, Istanbul, Turkey

1. Materials and equipment

Dimethylsulfoxide (DMSO), dimethylformamide (DMF), toluene, chloroform ($CHCl_3$), ethanol (EtOH), methanol (MeOH),acetone, dichloromethane, ethyl acetate, THF, diethyl ether, hexane, were purchased from Merck. Column chromatography was performed on alumina gel 60 (0.063–0.200 mm). FT-IR spectra were measured with a PerkinElmer Spectrum One Spectrometer. Absorption spectra in the UV-Visible region were obtained with a Shimadzu 2001 UV spectrophotometer. Elemental analyses were recorded with a Thermo Flash EA 1112 Series. Fluorescence spectra were measured using a Varian Eclipse spectrofluorometer using 1 cm path length cuvettes at room temperature. ^1H NMR spectra were recorded in deuterated chloroform ($CDCl_3$) solutions on a Varian 500 MHz spectrometer. Photo-irradiations were measured using a General Electric quartz line lamp (300W). A 600 nm glass cut off filter (Schott) and a water filter were used to filter off ultraviolet and infrared radiations respectively. An interference filter (Intor, 700 nm with a bandwidth of 40 nm) was additionally placed in the light path before the sample. Light intensities were measured with a POWER MAX5100 (Mol electron detector incorporated) power meter. Bandelin Ultrasonic RK 100 H was used for ultrasound irradiation. Mass spectra (ESI-MS) of Schiff bases were determined on a Finnigan LCQ Advantage MAX spectrometer andmass spectra of silicon phthalocyanines were determined on Bruker microflex LT MALDI-TOF MS. The instrument was operated in positive ion mode using am/z range of 50-3000. The capillary voltage of the ion source was set at 6000 V and the capillary exit at 190 V. The nebulizer gas flow was 1 bar and drying gas flow 8 mL/min (MALDI/MS).

2. Confirmation of structure

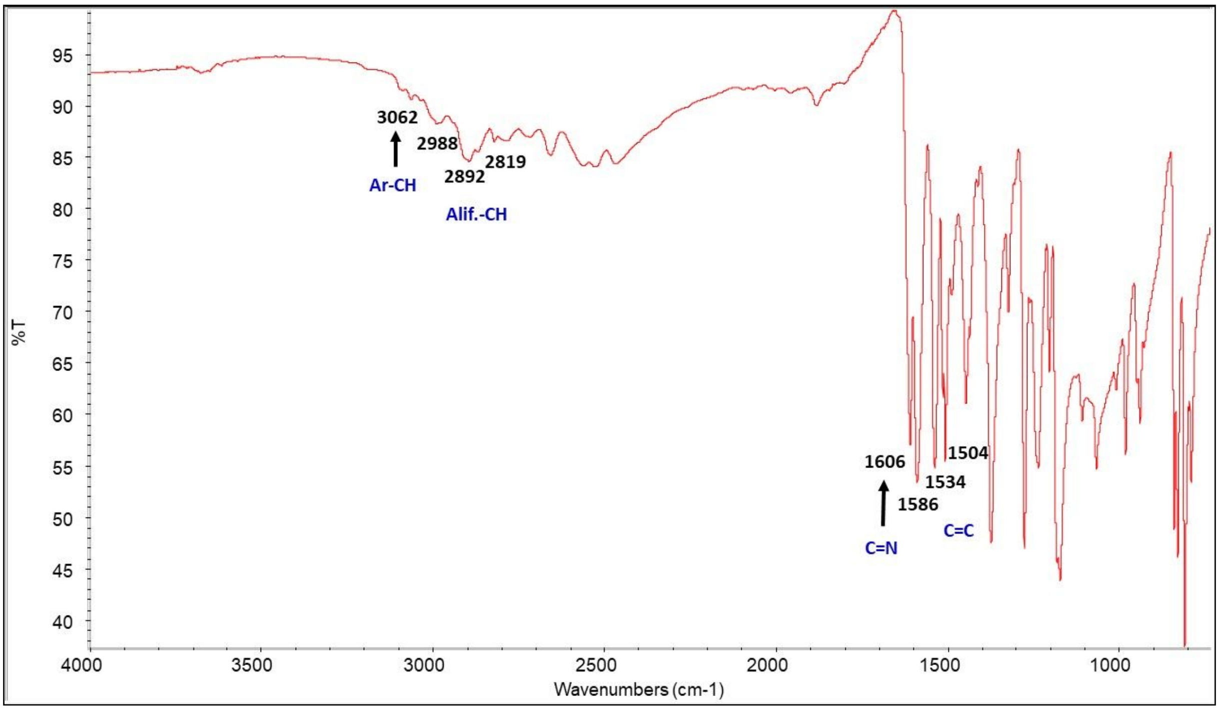

Figure.S1. FT-IR spectrum of compound **1a**.

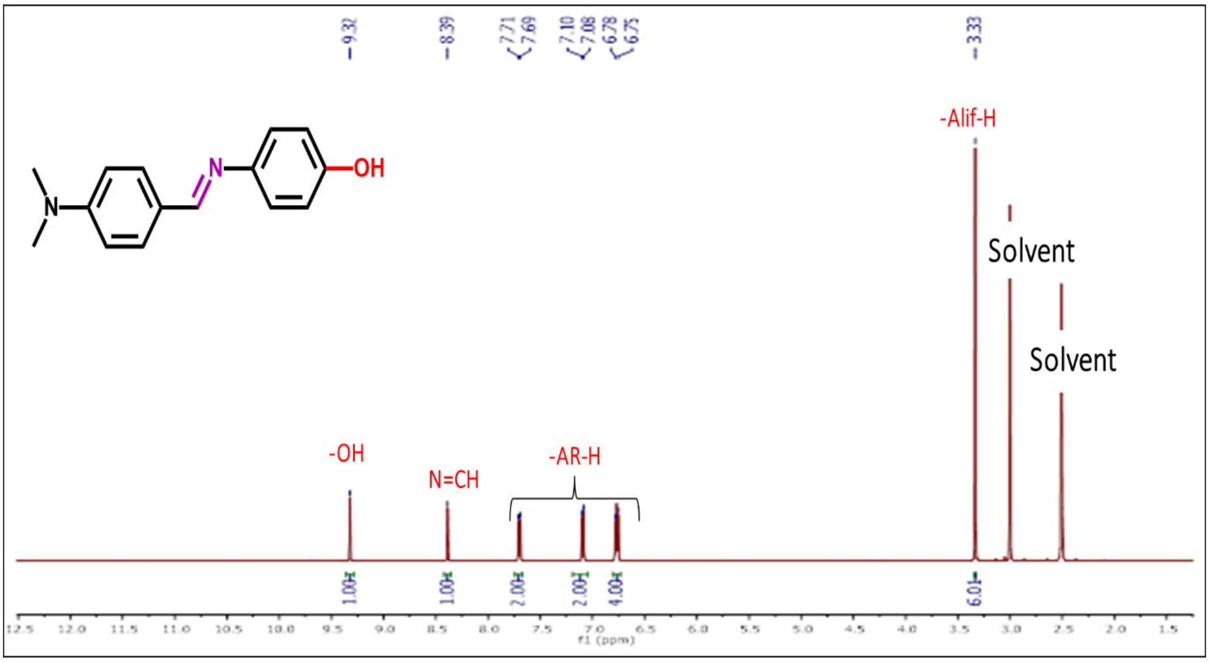

Figure.S2. ^{1}H-NMRspectrum of compound **1a.**

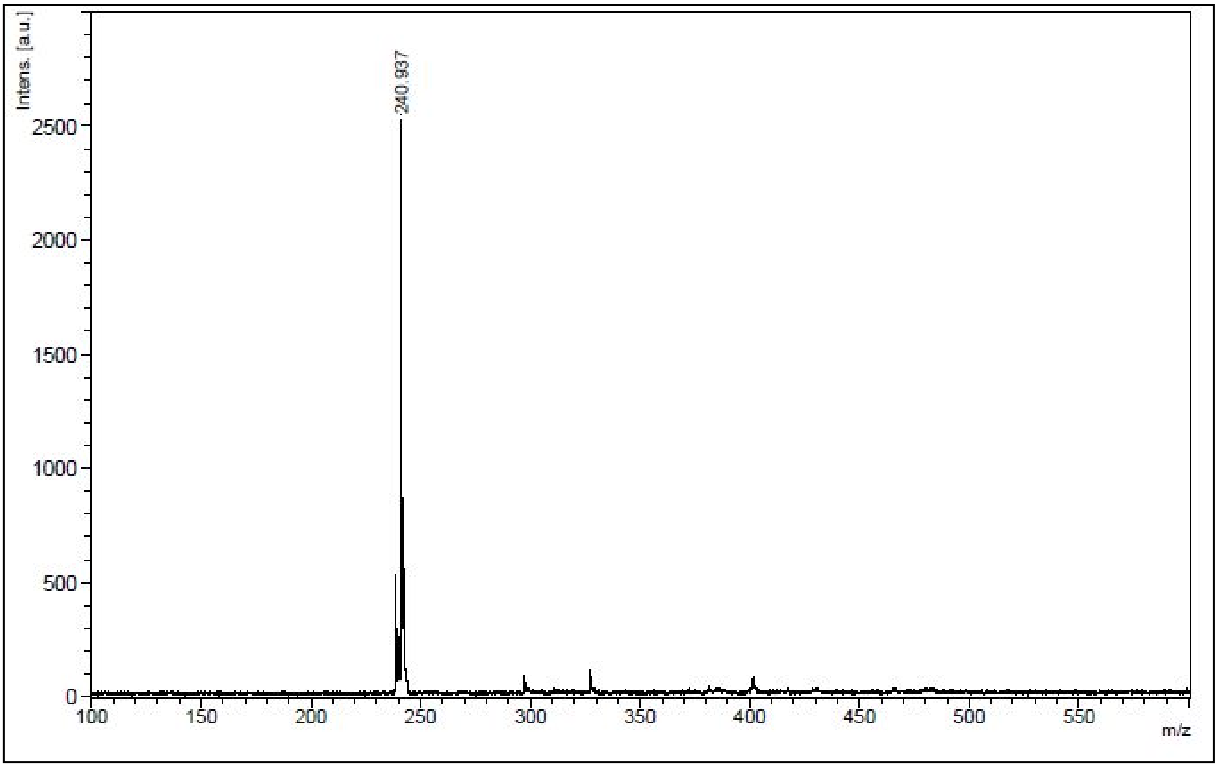

Figure.S3. Mass spectrum of compound **1a.**

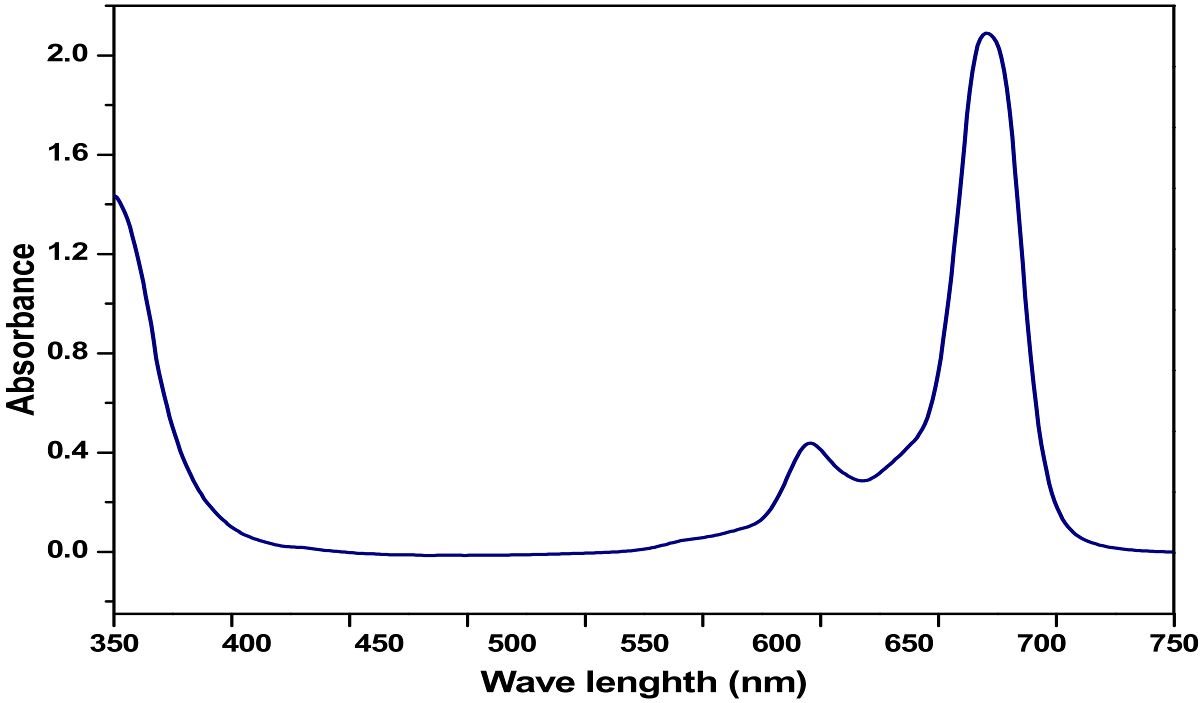

Figure.S4.UV-Vis spectrum of compound **Si1a.**

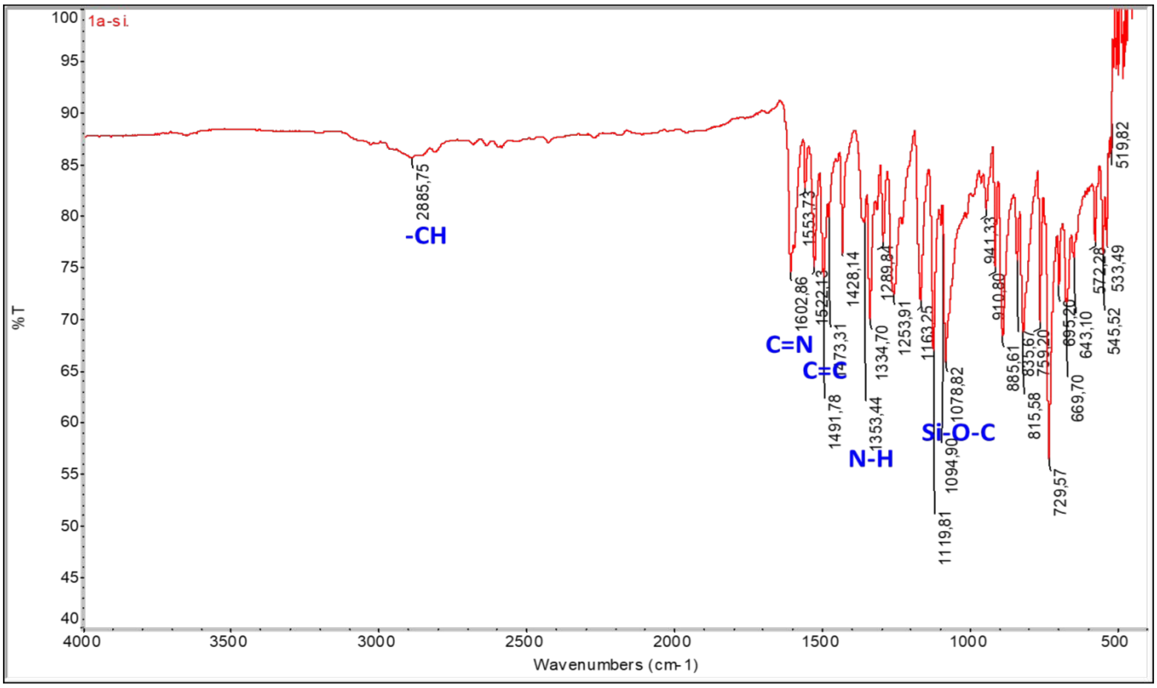

Figure.S5. FT-IR spectrum of compound **Si1a.**

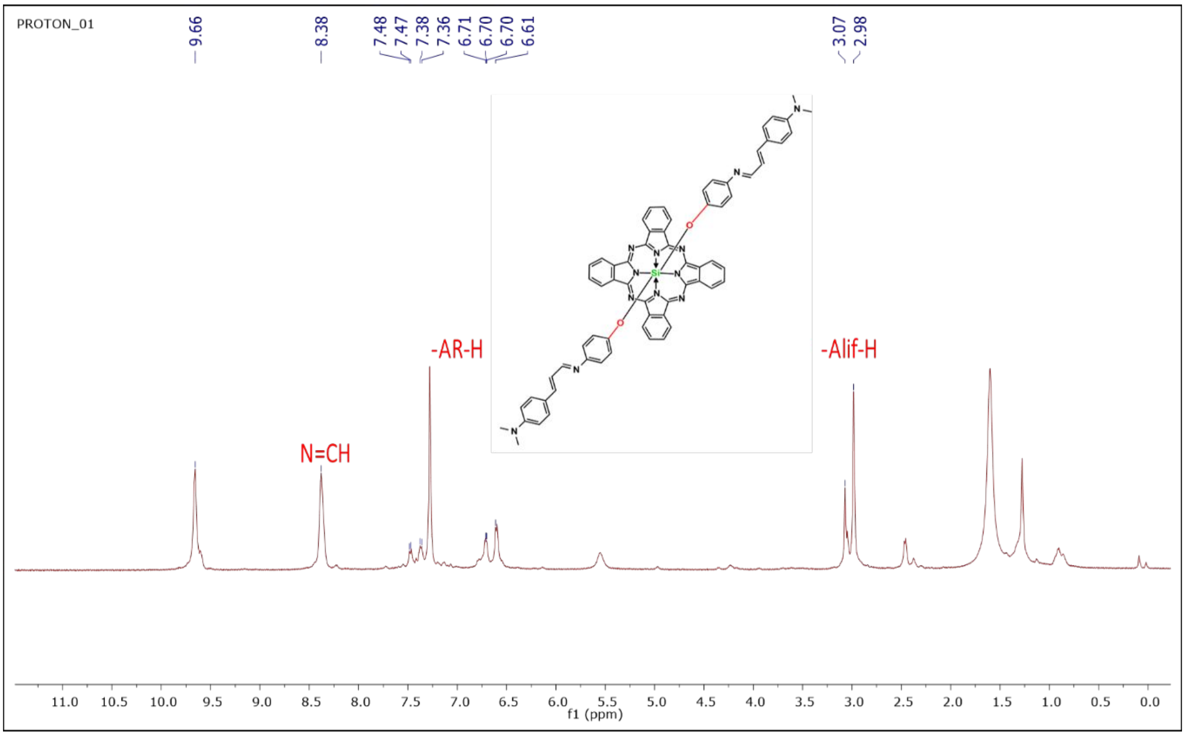

Figure.S6.^{1}H-NMRspectrum of compound **Si1a.**

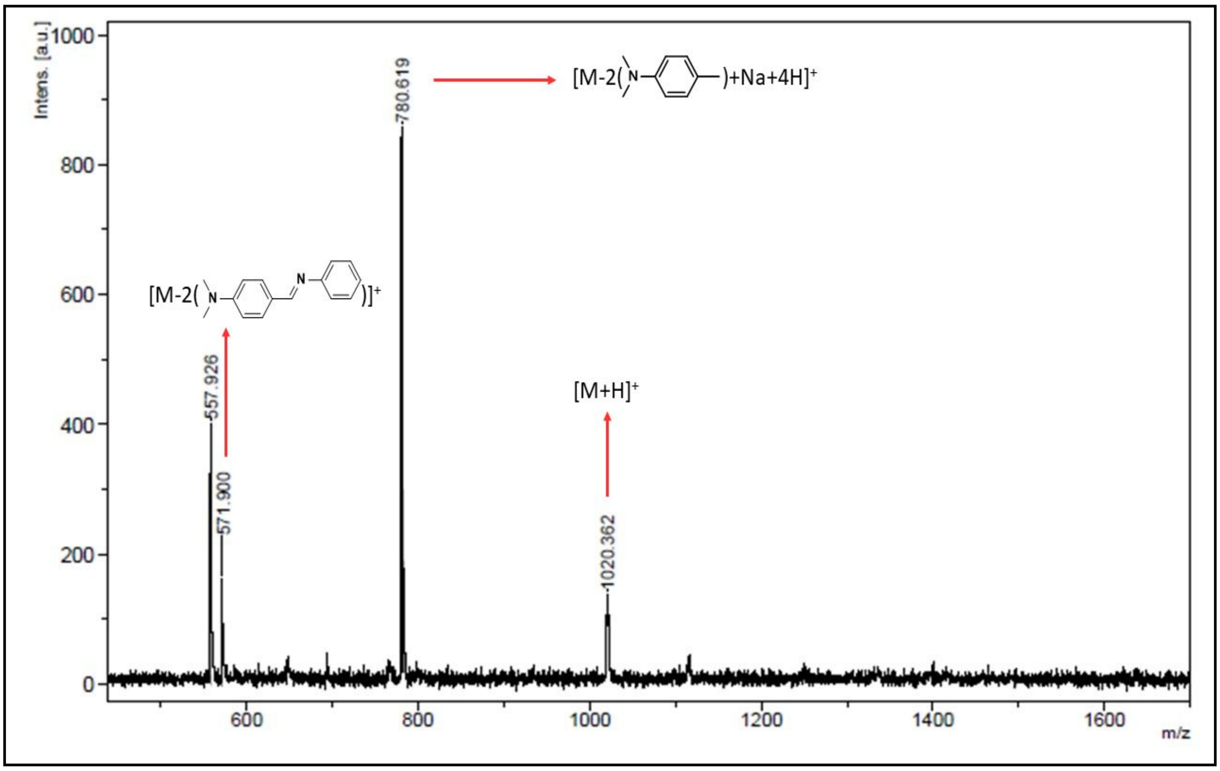

Figure.S7. Massspectrum of compound **Si1a.**

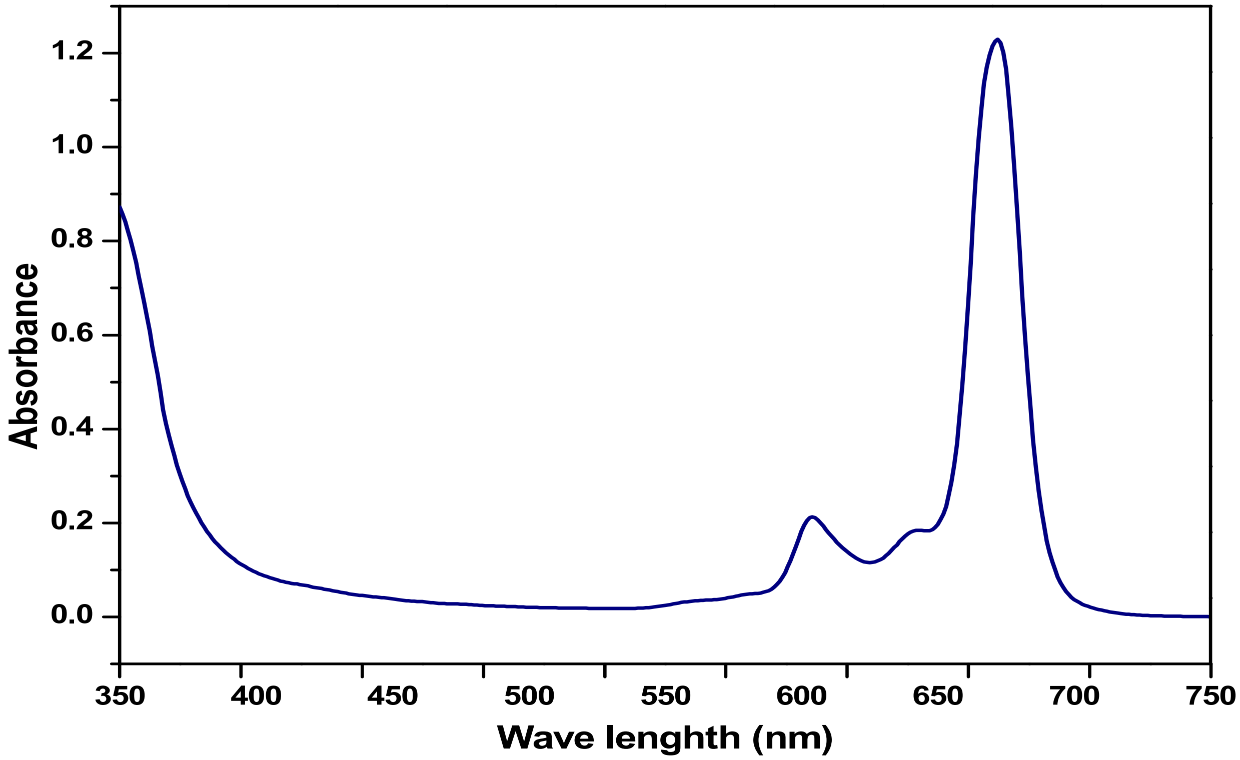

Figure S8.UV-Vis spectrum of compound **Q-Si1a.**

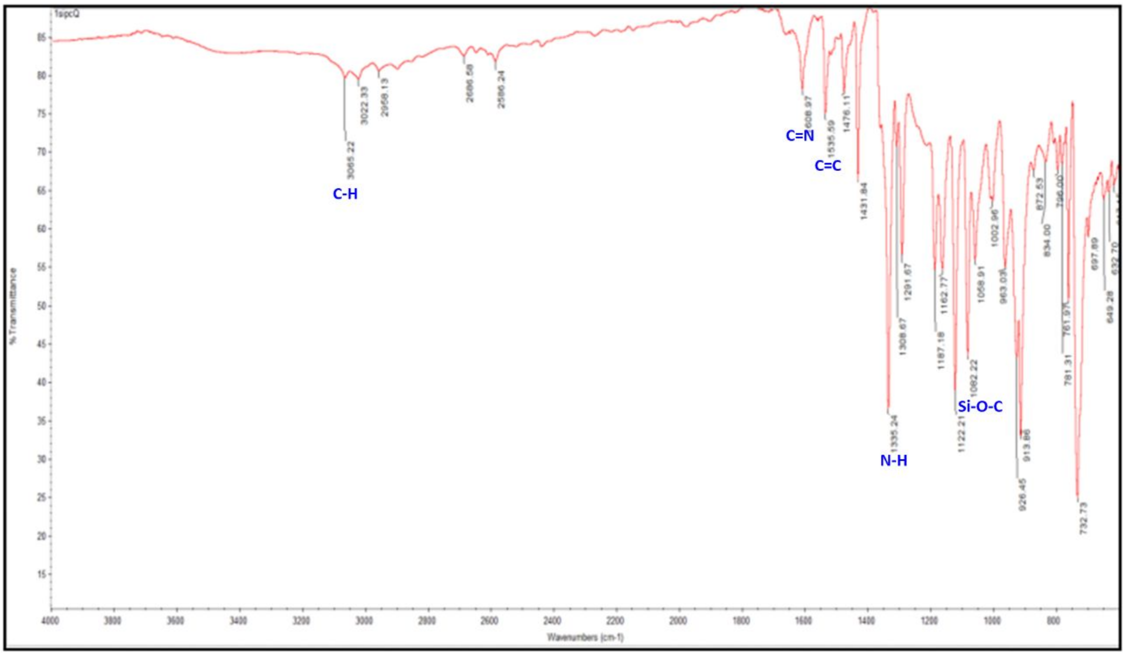

Figure.S9. FT-IR spectrum of compound Q-**Si1a.**

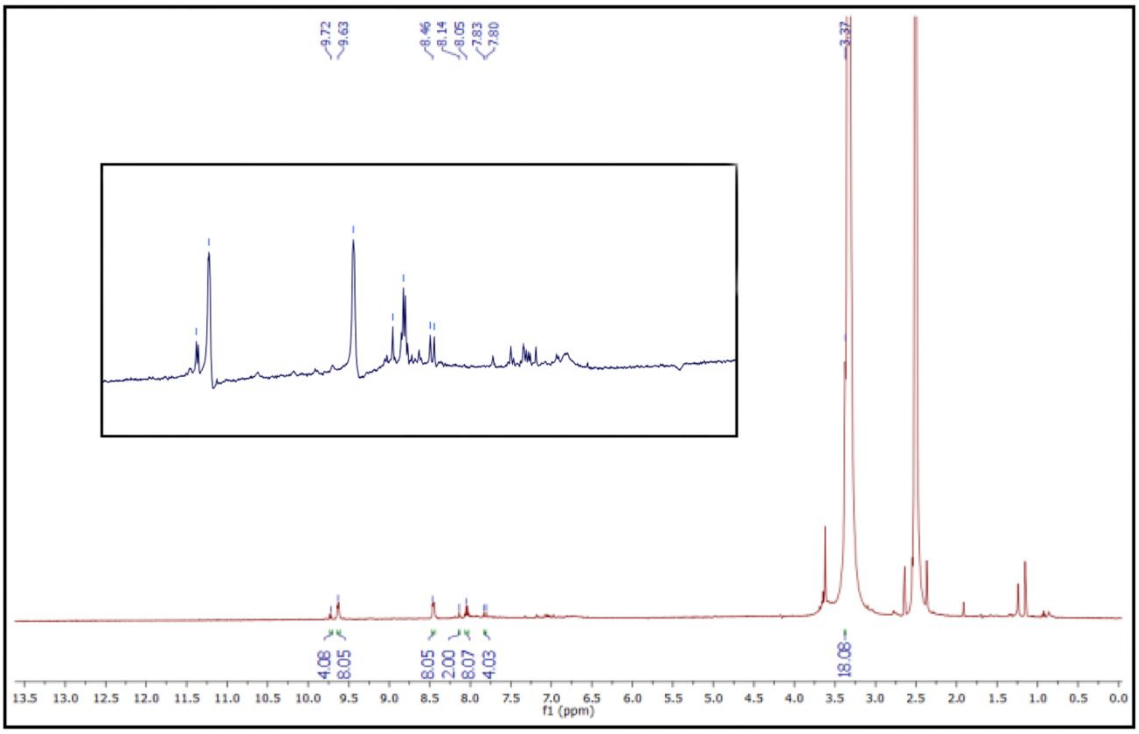

Figure.S10.^{1}H-NMRspectrum of compound **Q-Si1a.**

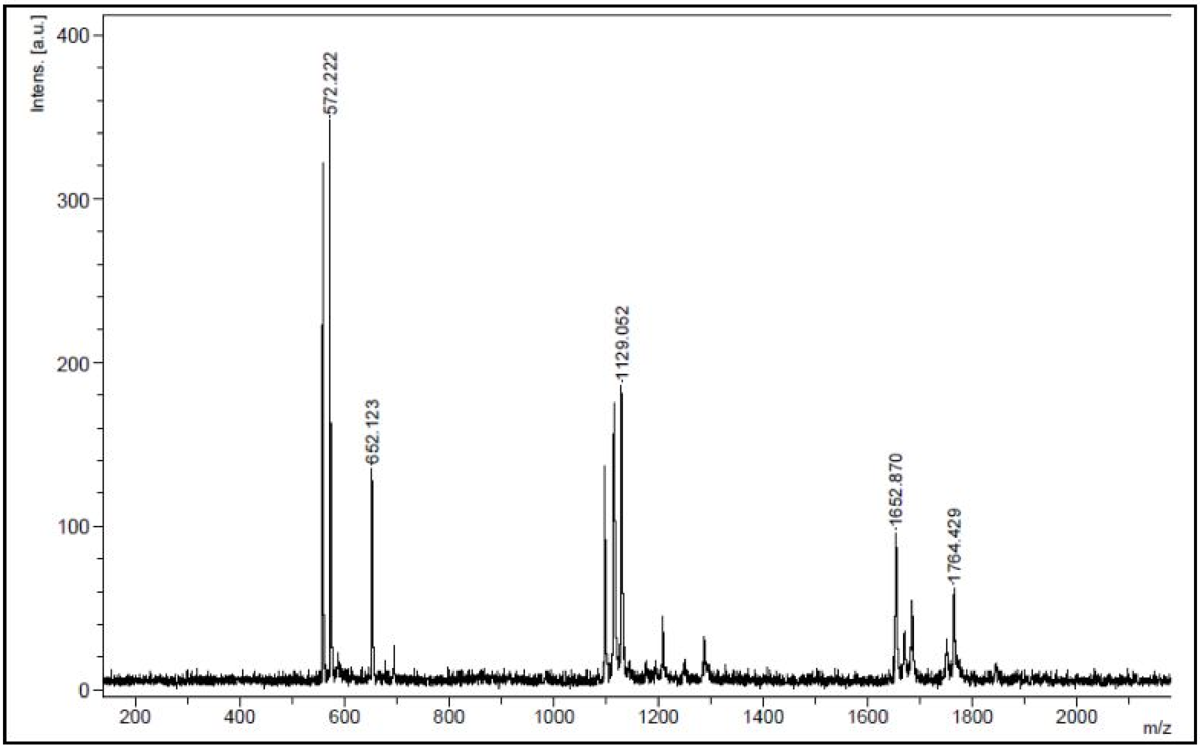

Figure.S11.MSspectrum of compound**Q-Si1a.**

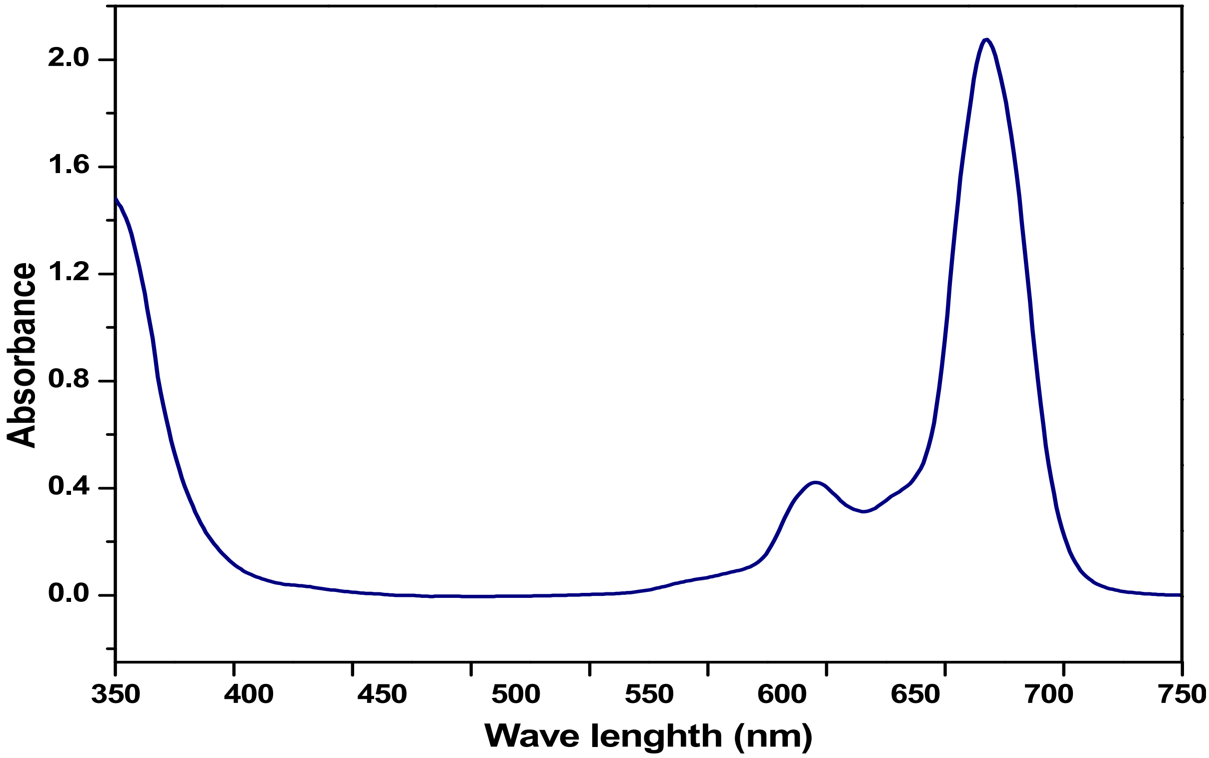

Figure.S12.UV-Vis spectrum of compound **S-Si1a.**

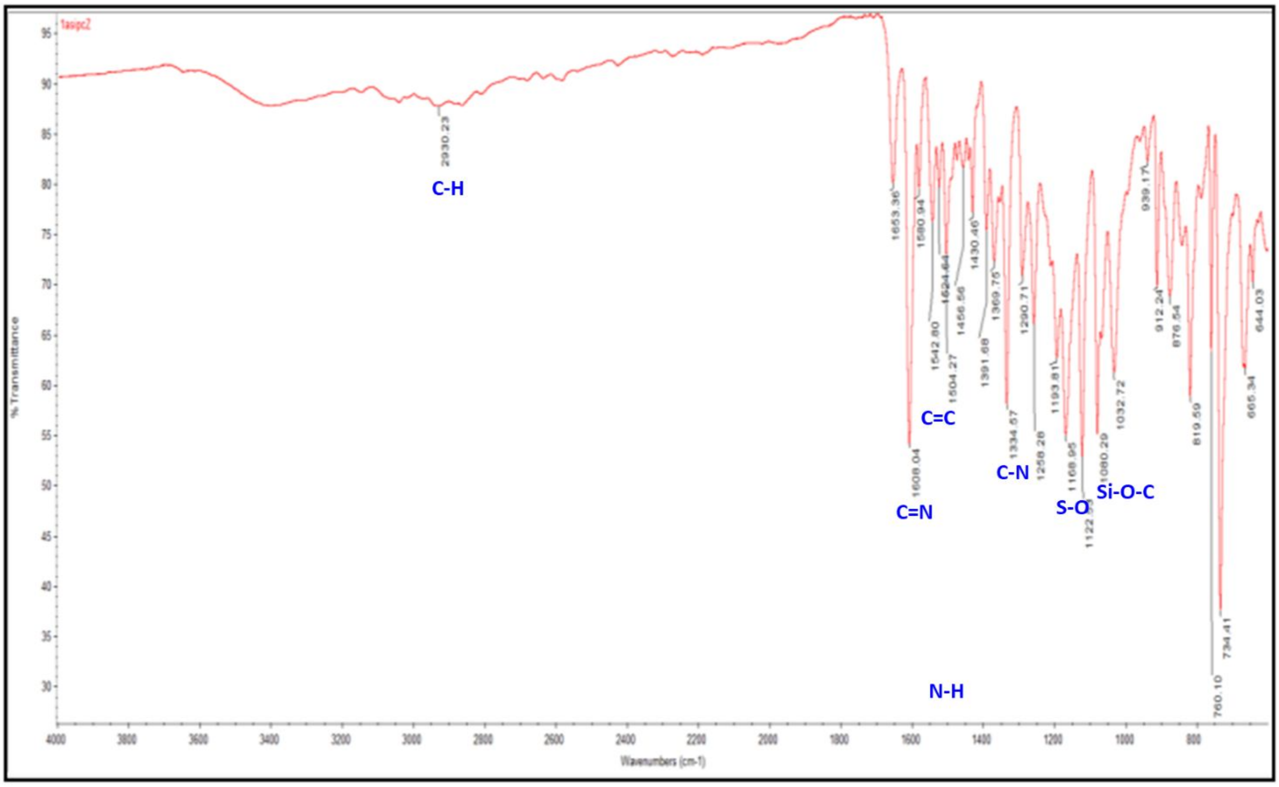

Figure.S13. FT-IR spectrum of compound **S-Si1a.**

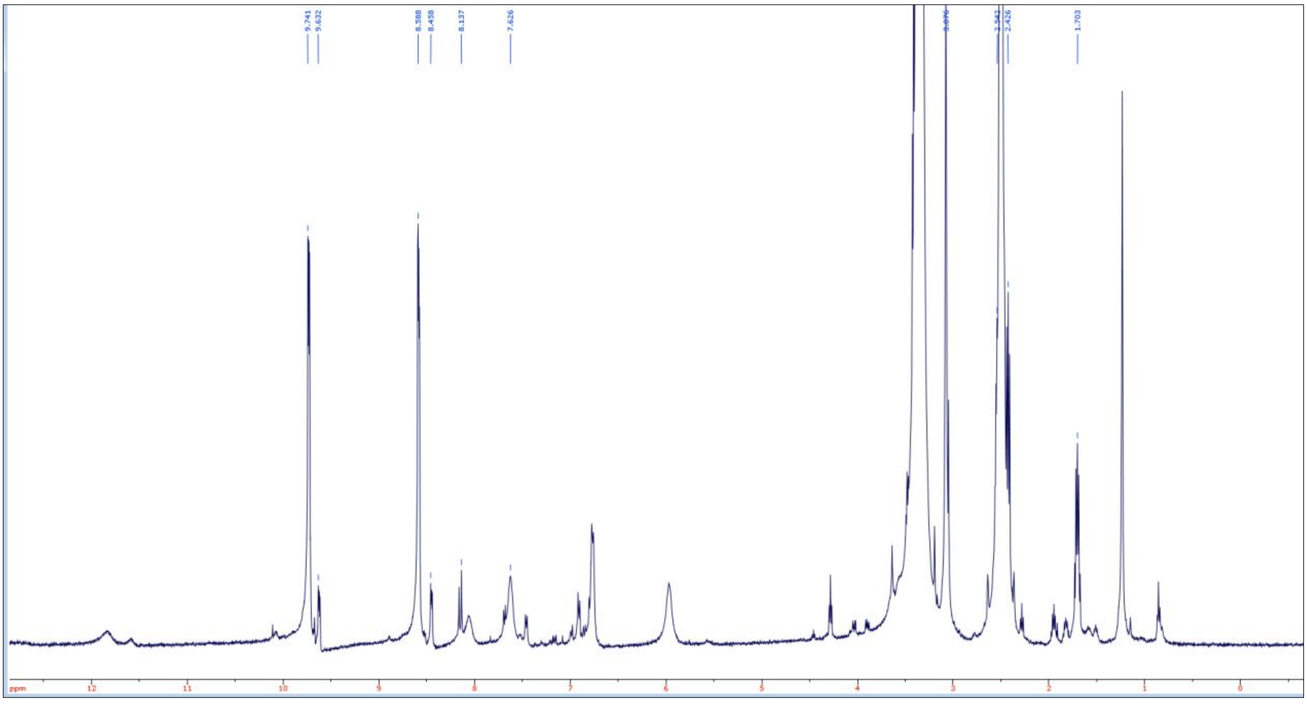

Figure.S14.^{1}H-NMRspectrum of compound **S-Si1a.**

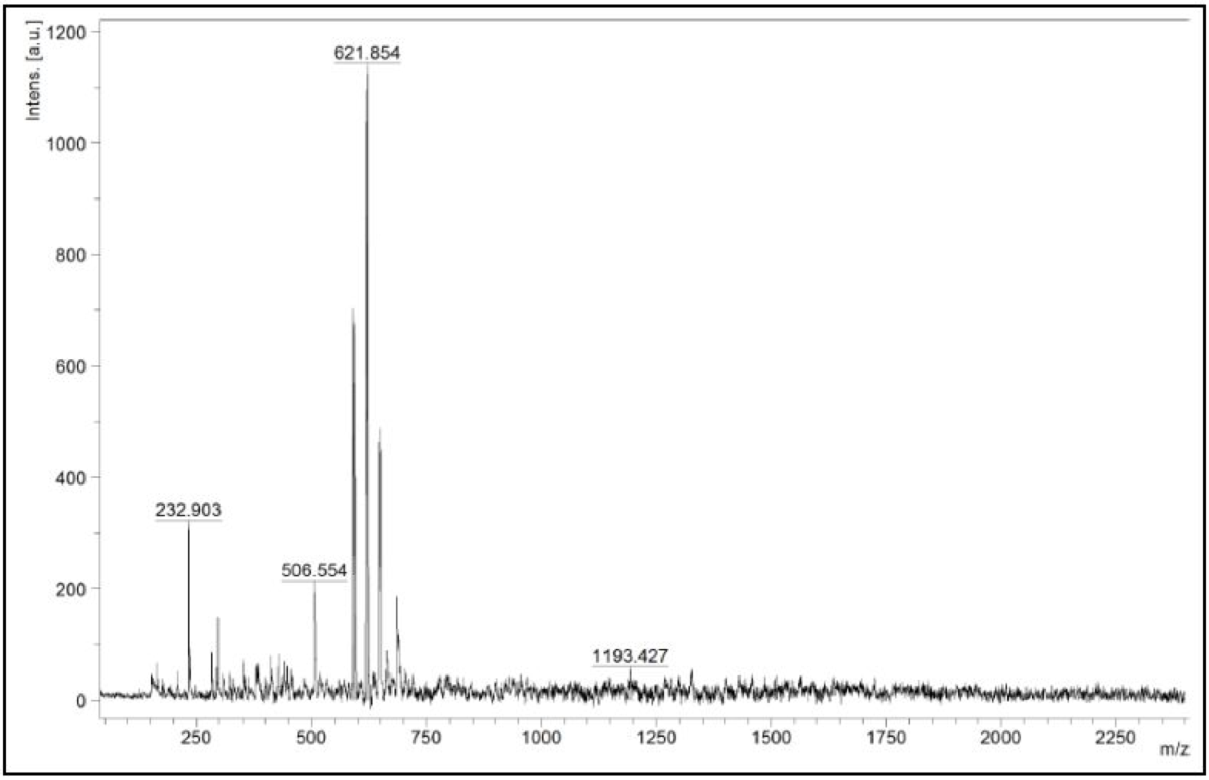

Figure.S15. Massspectrum of compound**S-Si1a.**

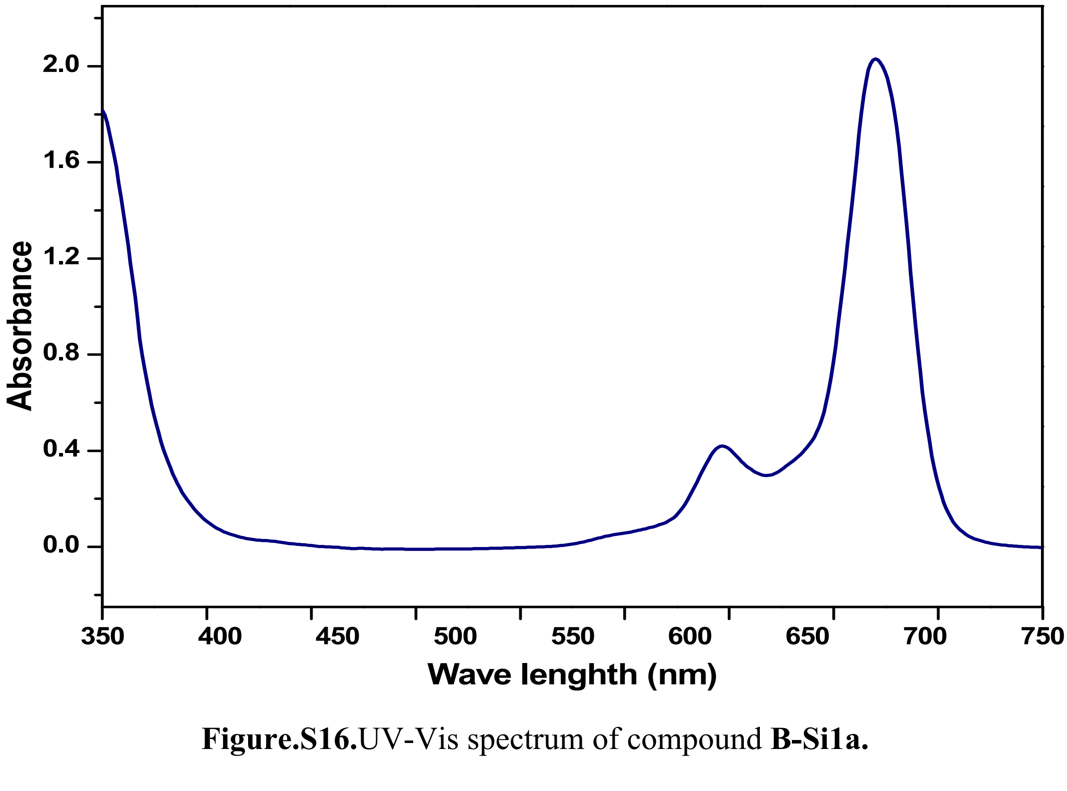

Figure.S16.UV-Vis spectrum of compound **B-Si1a.**

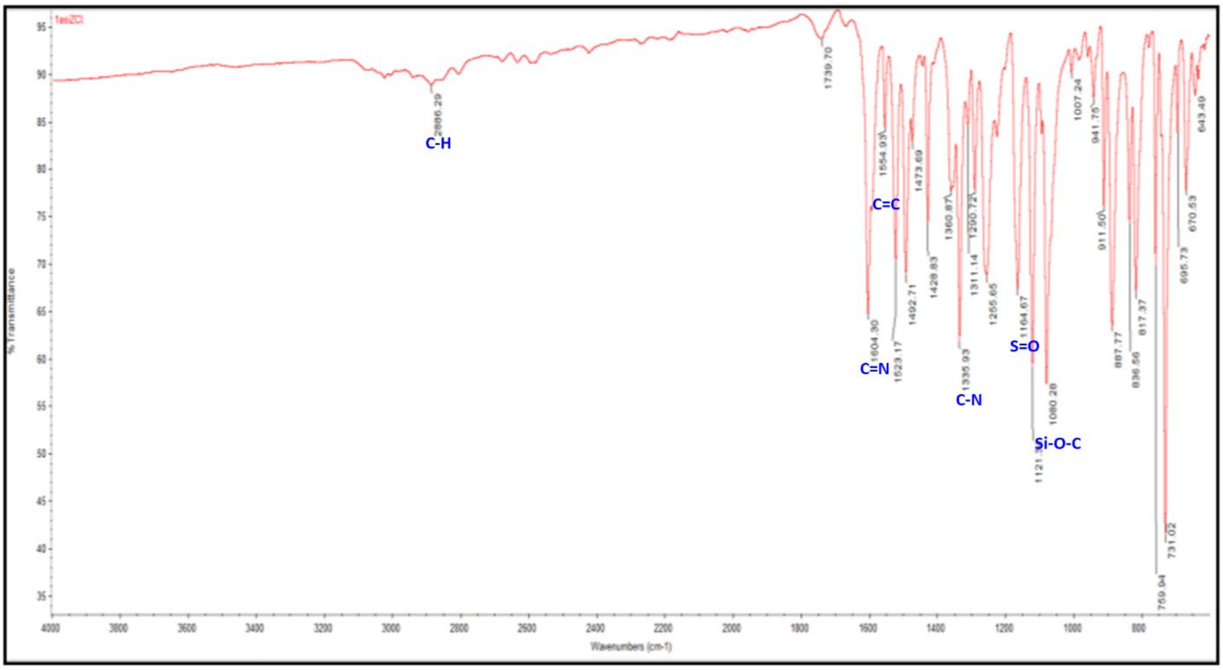

Figure.S17. FT-IR spectrum of compound **B-Si1a.**

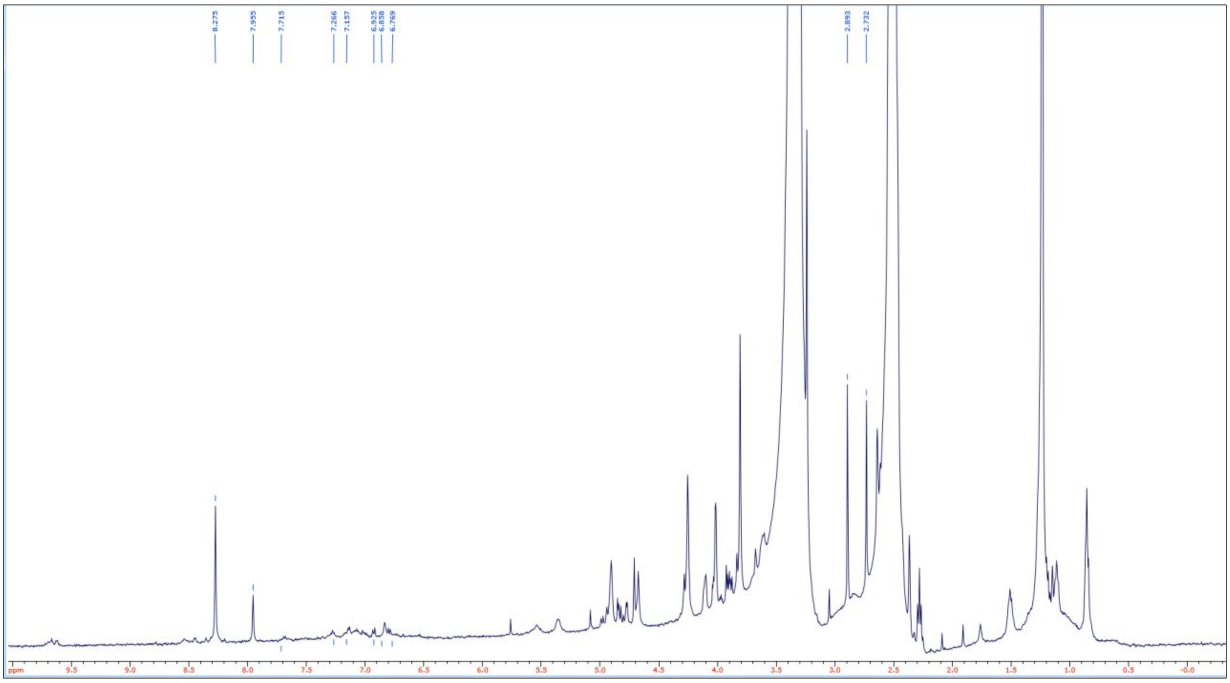

Figure.S18.¹H-NMRspectrum of compound **B-Si1a.**

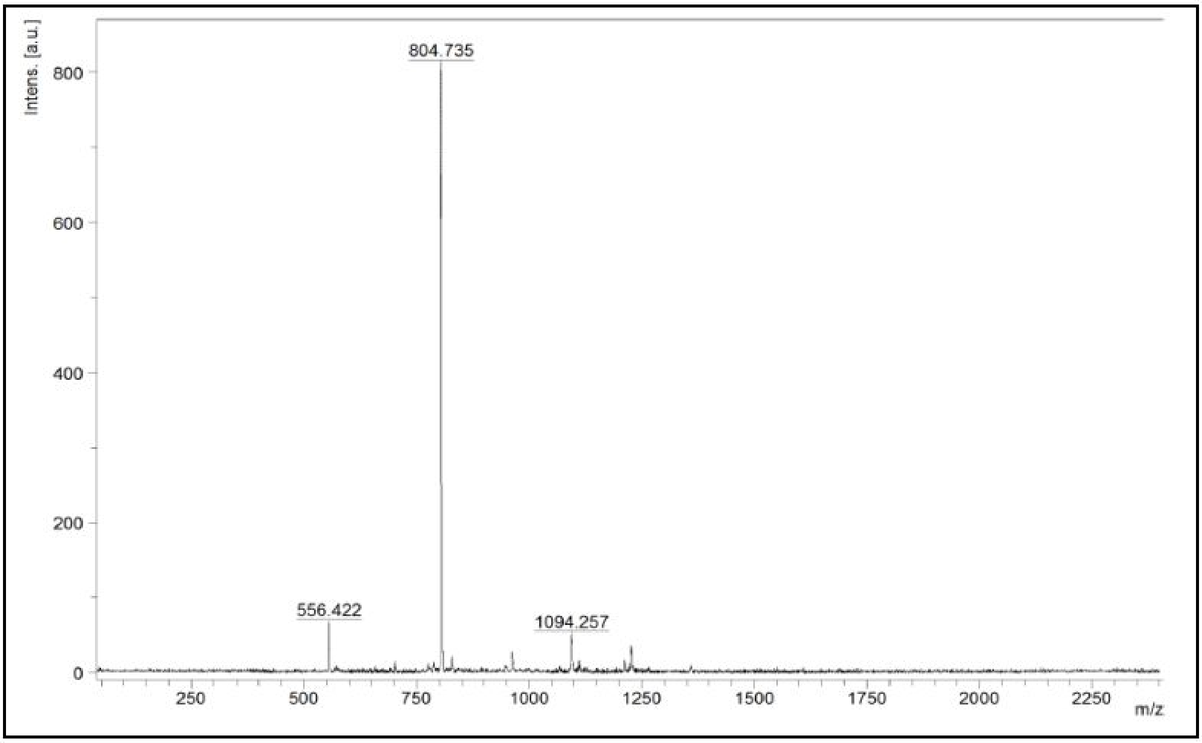

Figure.S19. Massspectrum of compound**B-Si1a.**

3. Singlet oxygen quantum yield

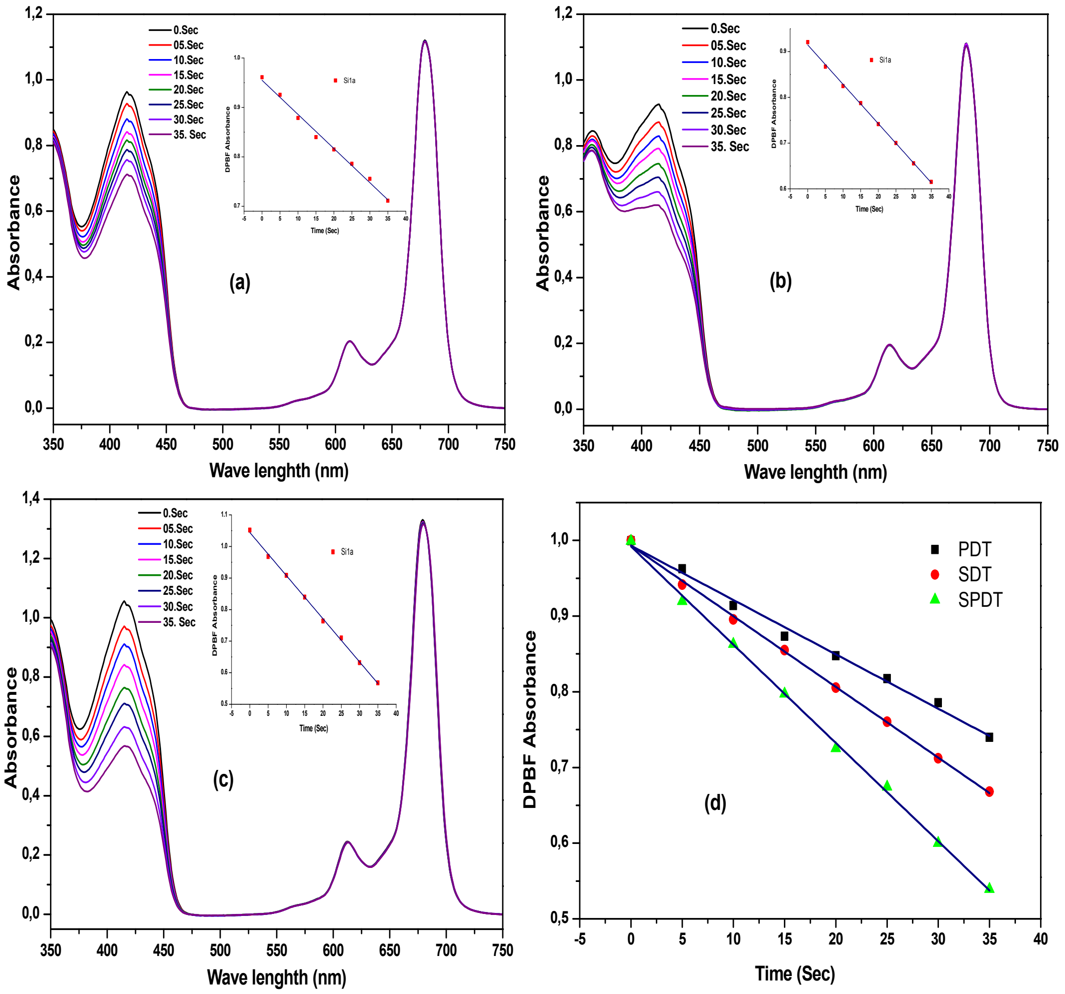

Figure S20. A typical spectrum for the determination of singlet oxygen quantum yield of the Si1a compound by (a) photochemical, (b) sonochemical, (c) sono-photochemical, and (d) DPBF concentration change.

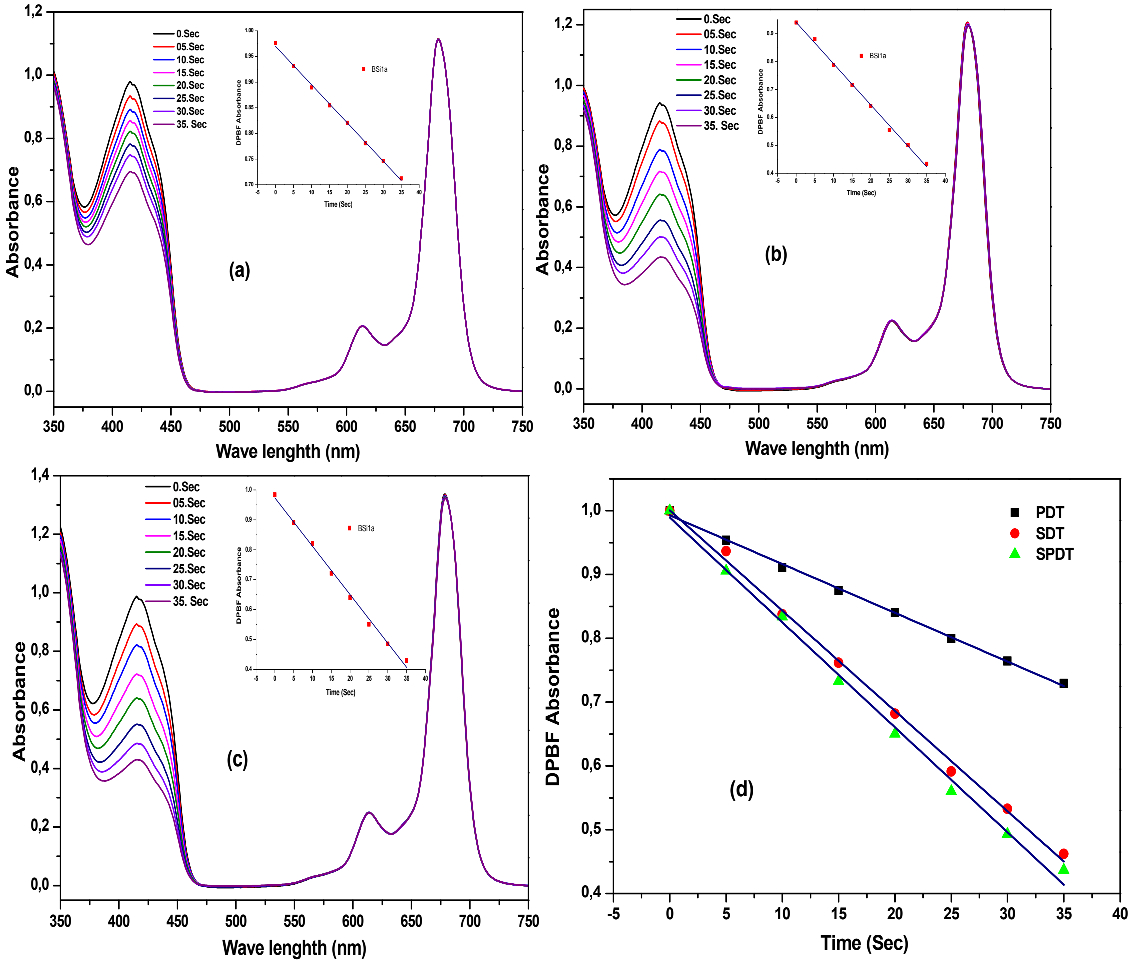

Figure S21. A typical spectrum for the determination of singlet oxygen quantum yield of the B-Si1acompound by (a)photochemical, (b)sonochemical, (c)sono-photochemical, and (d) DPBF concentration change.

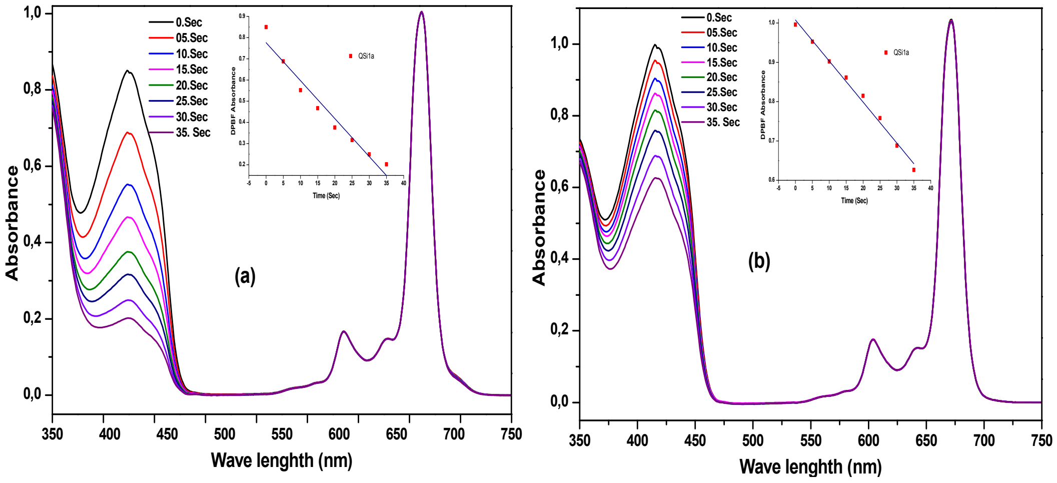

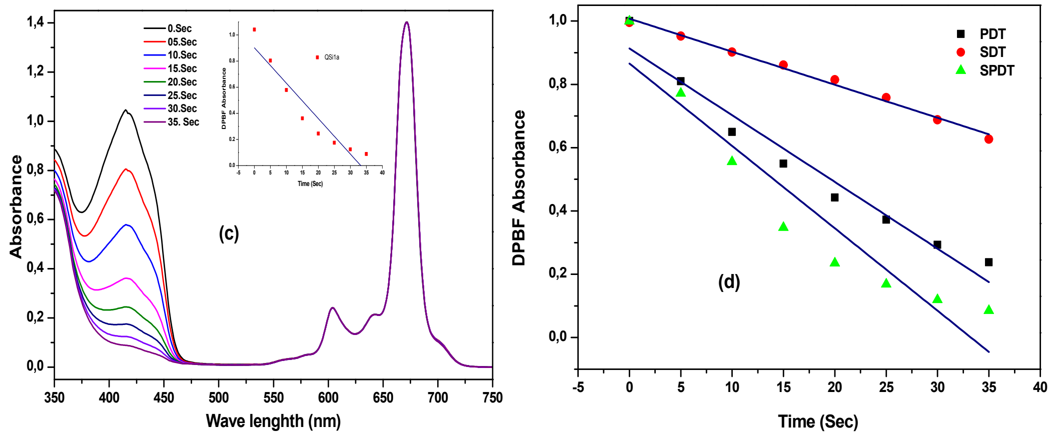

Figure S22. A typical spectrum for the determination of singlet oxygen quantum yield of the Q-Si1acompound by (a) photochemical, (b) sonochemical, (c) sono-photochemical,and (d) DPBF concentration change.

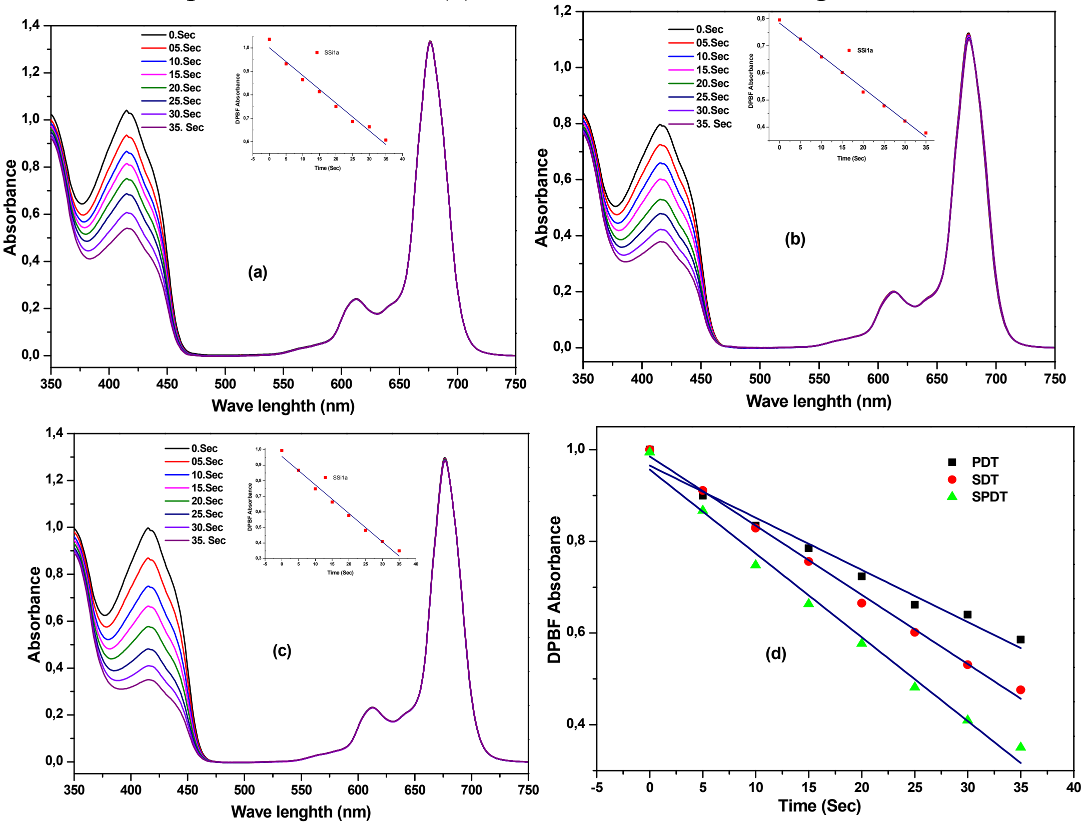

Figure S23. A typical spectrum for the determination of singlet oxygen quantum yield of S-Si1acompounds by (a) photochemical, (b) sonochemical, (c) sono-photochemical, and(d) DPBF concentration change.

4. Photodegradation quantum yield

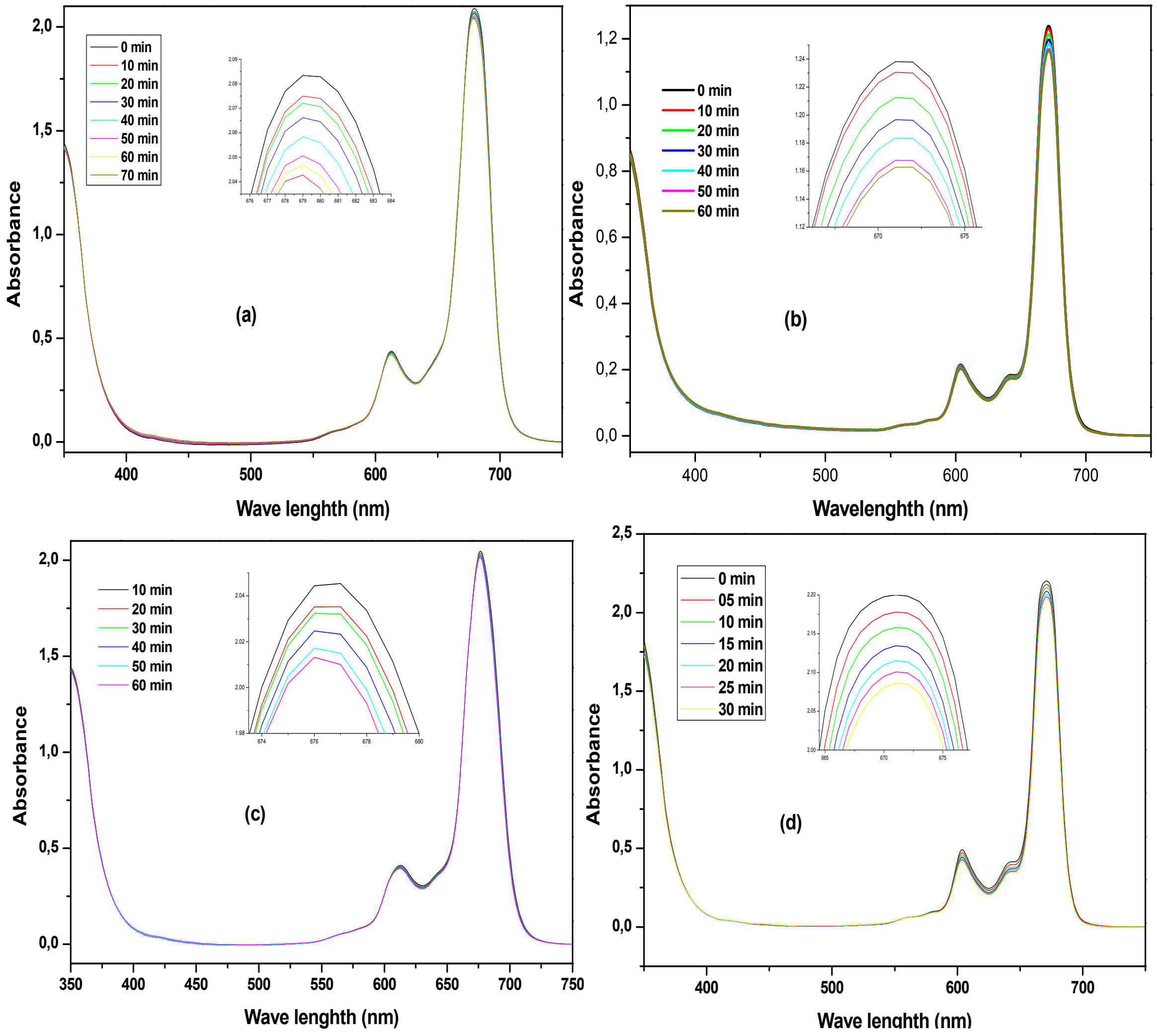

Figure S24. The spectrum of the determination of the photodegradation quantum yield of the compounds **Si1a**, **Q-Si1a**, **S-Si1a**, and **B-Si1a** in DMSO.

5. MTT cytotoxicity results

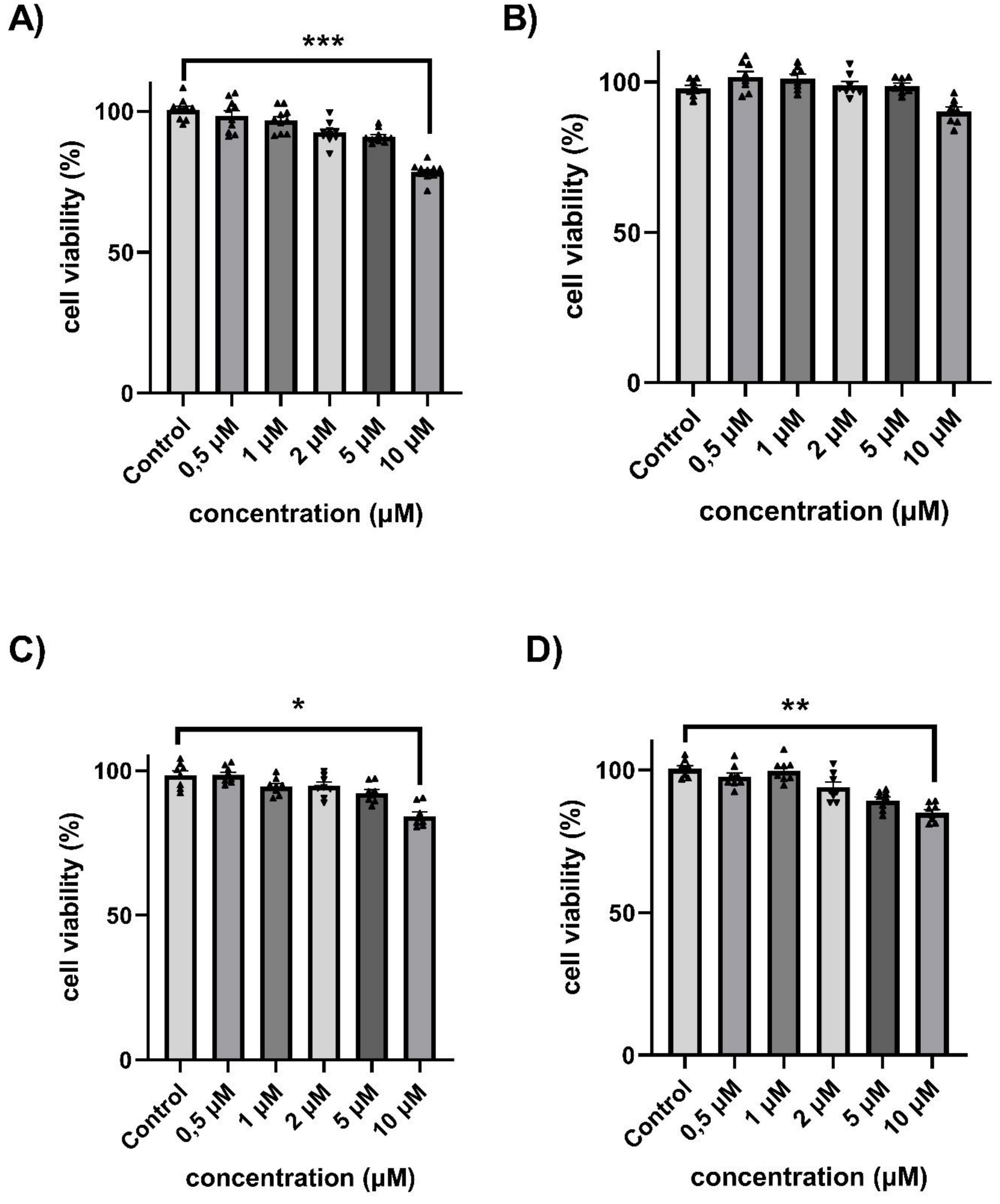

Figure S25. MTT cytotoxicity results, A) Si1a, B) B-Si1a, C) S-Si1a and D) Q-Si1a *p < 0.05, **p < 0.01, ***p < 0.001, and ****p < 0.0001.
